# Possible Existence of Lysosome-Like Organella within Mitochondria and Its Role in Mitochondrial Quality Control

**DOI:** 10.1371/journal.pone.0016054

**Published:** 2011-01-17

**Authors:** Yuji Miyamoto, Noriaki Kitamura, Yasuyuki Nakamura, Manabu Futamura, Takafumi Miyamoto, Masaki Yoshida, Masaya Ono, Shizuko Ichinose, Hirofumi Arakawa

**Affiliations:** 1 Cancer Medicine and Biophysics Division, National Cancer Center Research Institute, Tokyo, Japan; 2 Chemotherapy Division, National Cancer Center Research Institute, Tokyo, Japan; 3 Instrumental Analysis Research Center, Tokyo Medical and Dental University, Tokyo, Japan; University of Medicine and Dentistry of New Jersey, United States of America

## Abstract

The accumulation of unhealthy mitochondria results in mitochondrial dysfunction, which has been implicated in aging, cancer, and a variety of degenerative diseases. However, the mechanism by which mitochondrial quality is regulated remains unclear. Here, we show that Mieap, a novel p53-inducible protein, induces intramitochondrial lysosome-like organella that plays a critical role in mitochondrial quality control. *Mieap* expression is directly regulated by p53 and is frequently lost in human cancer as result of DNA methylation. Mieap dramatically induces the accumulation of lysosomal proteins within mitochondria and mitochondrial acidic condition without destroying the mitochondrial structure (designated MALM, for Mieap-induced accumulation of lysosome-like organelles within mitochondria) in response to mitochondrial damage. MALM was not related to canonical autophagy. MALM is involved in the degradation of oxidized mitochondrial proteins, leading to increased ATP synthesis and decreased reactive oxygen species generation. These results suggest that Mieap induces intramitochondrial lysosome-like organella that plays a critical role in mitochondrial quality control by eliminating oxidized mitochondrial proteins. Cancer cells might accumulate unhealthy mitochondria due to *p53* mutations and/or *Mieap* methylation, representing a potential cause of the Warburg effect.

## Introduction

In aerobic eukaryotic cells, the energy source is the mitochondria, which produce ATP by oxidative phosphorylation [1]. These cytoplasmic organelles are therefore essential for growth, division, and energy metabolism in aerobic eukaryotic cells. Each cell contains hundreds of mitochondria, and without these organelles, even cancer cells are unable to grow and survive *in vivo* [2]. Mitochondria also play a pivotal role in apoptosis [3]. The functional and physical interplay of the Bcl-2 family of proteins, including Bax and Bak, in the mitochondria promotes the opening of the outer membrane pore, leading to the release of cytochrome *c*, caspase activation, and apoptosis. Mitochondria are therefore central to two distinct cellular processes: cell survival and cell death [4]. As a consequence, deficits in mitochondrial function have been implicated in various human degenerative diseases, aging, and cancer [5], [6].

Mitochondria are also the primary source of reactive oxygen species (ROS) within the cell because of their importance in aerobic energy production by the respiratory chain. Even under normal conditions, mitochondria generally produce small amounts of ROS during respiratory energy production [7]. In addition, dysfunctional mitochondria produce much higher levels of ROS. This could be due to abnormal electron transfer by dysfunctional respiratory chain proteins; impaired ATP production by dysfunctional ATP synthase proteins; and/or the decreased supply of NADH resulting from dysfunctional TCA cycle proteins. The generated ROS also oxidize mitochondrial proteins, including the core proteins of energy production themselves, leading to a vicious cycle and the acceleration of mitochondrial dysfunction [7], [8]. Furthermore, the ROS generated by dysfunctional mitochondria oxidize and damage intracellular DNA, RNA, lipids, and proteins, thereby leading to a variety of cellular dysfunctions [5], [6]. Therefore, the efficient elimination of the oxidized mitochondrial proteins and the resulting prevention of mitochondrial ROS generation appear to be one of the critical mechanisms of mitochondrial quality control.

Although the quality control of mitochondria is important for maintaining the steady and healthy state of our bodies [9], the mechanisms involved remains to be elucidated. A possible quality control mechanism is the degradation of the entire mitochondrion, known as mitophagy [10]. In yeast, macroautophagy and/or microautophagy has been suggested to play an important role in mitophagy to eliminate damaged and unhealthy mitochondria, thereby maintaining the quality of the organelle [11]. Additionally, the degradation and elimination of damaged proteins in mitochondria also plays a pivotal role in maintaining the healthy status of mitochondria [12]. Previous studies have reported the existence of several mitochondrial proteases that play a critical role in the degradation of mitochondrial proteins [13]. Moreover, several mitochondrial proteins have been suggested as substrates for these proteases [14], [15]. However, the mechanism for the degradation of most mitochondrial proteins remains to be elucidated.

Lysosomes are membrane-surrounded cytoplasmic organelles involved in intracellular protein degradation [16], [17]. The lysosomal compartment contains nearly 50 acid-dependent hydrolases (pH ∼4 to 5) such as proteases, lipases, and glycosidases within its lumen [16]. Therefore, lysosomes act as kind of “stomach” in the cells and plays a crucial role in the degradation of almost all content within the cell, including protein, DNA, organelle, and bacteria. Because of its powerful ability to degrade all kinds of materials, the process must be strictly controlled in order to execute its full function. Otherwise, disregulated digestion by lysosome within the cell causes harmful effects on the cellular organelles and proteins, leading to cell death. Both endogenous and exogenous macromolecules are delivered to lysosomes through the biosynthetic (macroautophagy) and endocytic (endocytosis) pathways, respectively [18]. Lysosomes also engulf organelles (microautophagy) [18]. In addition, lysosomes can specifically and directly degrade the proteins transported from cytosol (chaperon-mediated autophagy: CMA) [19]. Currently, other pathways have not been identified for lysosomal degradation of the proteins and organelle within the cell.

Aerobic glycolysis is increased in many cancer cells, which is known as the Warburg effect [20]. Although the mechanism underlying this phenomenon remains largely unclear, accumulating evidence suggests that mitochondrial dysfunction at least partially contributes. Cancer cells accumulate mitochondrial DNA mutations, leading to impaired mitochondrial respiration and ATP production [21]–[23]. Furthermore, the tumor suppressor p53 regulates the transcription of mitochondrial respiratory chain proteins, including SCO1 [24] and CABC1 [25]. As the *p53* gene is frequently mutated in cancer cells, the expression of these proteins is downregulated, leading to defects in mitochondrial respiration [26]. The evidence suggests that the mitochondria of cancer cells become dysfunctional, leading to impaired respiratory ATP production and upregulated aerobic glycolysis.

Here we report a novel mechanism of mitochondrial quality control. Mieap, a novel p53-inducible protein, induced intramitochondrial lysosome-like organelles to eliminate the oxidized mitochondrial proteins without destroying the mitochondrial structure. This phenomenon was not related to canonical autophagy. The function was regulated by tumor suppressor p53. Frequent inactivation of the p53-Mieap pathway in cancer cells could represent a potential cause of the Warburg effect.

## Results

### Identification of *Mieap* as a p53-target gene and its inactivation in human cancer

To identify p53-regulated genes, we screened for p53-inducible genes using microarrays, as reported previously [27]. The expression of *Mieap* was notably induced by exogenous and endogenous p53 in several cell lines (data not shown). *Mieap*, also designated *Spetex-1* in rats, has been reported to be expressed in spermatocytes at the late stage of spermatogenesis, although its function is unknown [28]. Therefore, we further analyzed the *Mieap* gene and its protein product. Mieap did not show significant amino acid sequence similarity to other well-characterized proteins in the BLAST database, but it does contain two coiled-coil motifs. Two forms of the Mieap protein could be expressed using alternative splice sites: Mieap-α and Mieap-β (DDBJ accession numbers AB465501 and AB465502, respectively).

**Figure 1:**
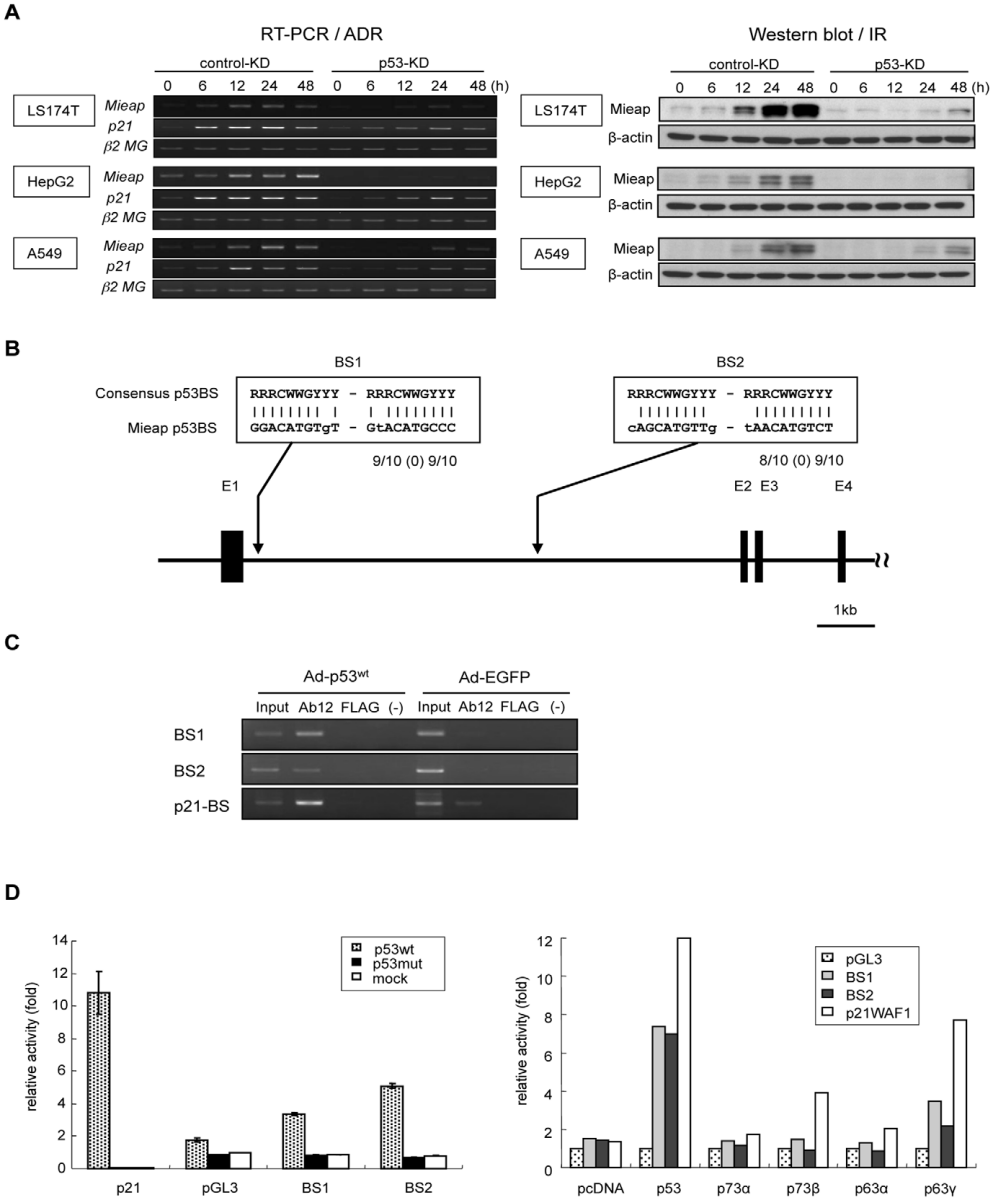
Identification of Mieap as a p53-target. (A) p53-dependency of Mieap expression. The control and p53-KD cells of LS174T, HepG2 and A549 were subjected to ADR or ionizing radiation (IR), and Mieap expression was examined by RT-PCR or western blot analysis at the indicated times. β2-MG and β-actin were used as loading controls. (B) p53-binding sequences of the *Mieap* gene. Two p53-binding sequences (BS1 and BS2) were identified in intron 1 of *Mieap*. (C) Binding of p53 with BS1 and BS2 of *Mieap*. Two p53-binding sites (BS1 and BS2) were identified in intron 1 of *Mieap*. A ChIP assay was performed on the DNA protein complex, which was immunoprecipitated by anti-p53 antibody (Ab12), anti-FLAG antibody (FLAG), or no antibody (-) from H1299 cells infected with Ad-p53 or Ad-EGFP at an MOI of 30. p21/WAF1 was used as a positive control. (D) p53-dependent transcriptional activity of BS1 and BS2. Heterologous reporter plasmid containing BS1 or BS2 was co-transfected with plasmid designed to express wild-type p53 (p53wt), mutant p53 (p53mut) or no p53 (mock) into H1299 cells. The heterologous reporter plasmid containing BS1 or BS2 was also co-transfected with the plasmid designed to express p53wt, p73α, p73β, p63α, p63γ or no p53 (mock) into H1299 cells. The luciferase activity 24 h after transfection is shown.

*Mieap* expression was induced in response to DNA damage in various *p53*–wild-type cancer cell lines (LS174T-Cont, HepG2-Cont, and A549-Cont), but not in the corresponding p53-knockdown (KD) isogenic cell lines (LS174T-p53-KD, HepG2-p53-KD, and A549-p53-KD; Figure 1A). We identified two possible p53-binding sequences, BS1 and BS2, in intron 1 of *Mieap* (Figure 1B). Using chromatin-immunoprecipitation (ChIP), the DNA fragment containing BS1 or BS2 could be immunoprecipitated with an anti-p53 antibody (Figure 1C). Moreover, a heterologous reporter assay showed that the transcriptional activity of a luciferase reporter plasmid containing BS1 or BS2 was enhanced by wild-type p53 but not by mutant p53, indicating that BS1 and BS2 are p53-responsive sequences of *Mieap* (Figure 1D). In addition, the activity appeared to be specific to p53, as p63 or p73 did not affect expression (Figure 1D). These findings suggest that *Mieap* is a *bona fide* target gene of the tumor suppressor p53.

**Figure 2:**
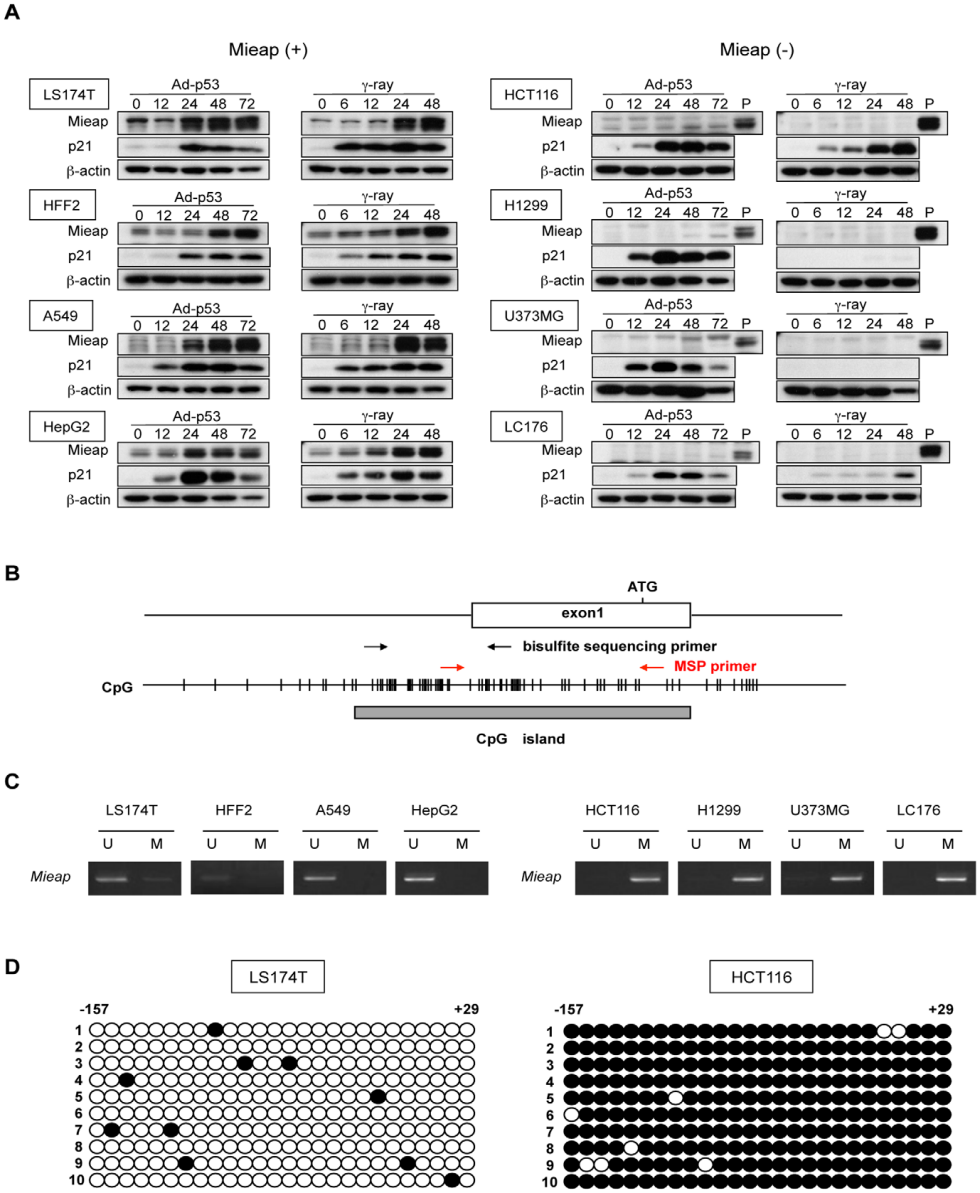
Mieap is frequently inactivated by methylation in human cancer. (A) Mieap expression was frequently downregulated in human cancers. Mieap protein expression in seven human cancer cell lines (LS174T, A549, HepG2, HCT116, H1299, U373MG and LC176) and one normal cell line (HFF2) were detected by western blot analysis. Proteins were isolated at the indicated times from cells infected with Ad-p53 or treated by IR, and then subjected to western blot analysis. Lane “P” shows Mieap protein expression in LS174T cells 48 h after IR as a positive control. p21/WAF1 was used as a positive control for a p53-target gene, and β-actin was used as a loading control. (B) Primers for MSP and bisulphite sequencing are located in the promoter region of *Mieap*. (C) MSP analysis of the *Mieap* promoter. Promoter *Mieap* was methylated in all four Mieap^−^ cell lines (HCT116, H1299, U373MG and LC176) but not in the four Mieap^+^ cell lines (LS174T, HFF2, A549 and HepG2). (D) Bisulphite sequencing analysis of the *Mieap* promoter. Bisulphite sequencing indicated that the promoter of Mieap in HCT116, but not LS174T, was largely methylated.

During the analysis of *Mieap* expression, we noticed that *Mieap* expression was frequently absent in human cancer cell lines. *Mieap* expression was primarily dependent on the p53 status of the cell following DNA damage but was lost in two cancer cell lines with wild-type p53: HCT116 (colorectal cancer) and LC176 (lung cancer) (Figure 2A). Moreover, *Mieap* expression was not rescued by the overexpression of exogenous *p53* in two *p53*-mutant cancer cell lines, H1299 (lung cancer) and U373MG (glioblastoma), or in the two *p53*–wild-type cancer cell lines (HCT116 and LC176) (Figure 2A). Therefore, we assessed the methylation status of the *Mieap* gene in these cancer cell lines by performing methylation-specific PCR (MSP) (Figure 2B). The *Mieap* promoter was methylated in all cell lines in which *Mieap* expression was repressed (Figure 2A and 2C). In contrast, the *Mieap* promoter was unmethylated in all cell lines in which *Mieap* expression was detected (Figure 2A and 2C). This was confirmed by the methylation sequences of two unmethylated cancer cell lines, LS174T (Figure 2D) and HepG2 (Figure S1A), and two methylated cancer cell lines, HCT116 (Figure 2D) and U373MG (Figure S1A), and by a demethylation experiment in the HCT116 cell line with azacytidine (Aza-C) treatment, which reactivated the transcription of *Mieap* (Figure S1B).

We examined the methylation status and expression level of *Mieap* in 10 additional cancer cell lines. *Mieap* expression was downregulated in over 60% of the cancer cell lines; the *Mieap* promoter was methylated in all of these (Figure S2). Therefore, methylation of the *Mieap* promoter could be correlated with the repression of *Mieap* expression. This suggests that *Mieap* is frequently inactivated by DNA methylation in human cancers.

### Mieap induces accumulation of lysosomal proteins and acidic condition in mitochondria

To investigate Mieap function, we generated an adenovirus vector to express the full-length Mieap-α protein (Ad-Mieap). To examine the subcellular localization of the Mieap protein, we performed immunofluorescence (IF) analysis with an anti-Mieap antibody. Using the Ad-Mieap vector, we ectopically expressed the low level of the Mieap protein by infecting the cells with Ad-Mieap at a multiplicity of infection (MOI) of 5, which is nearly a minimum dose of adenovirus. The expression pattern of Mieap in one *Mieap*-unmethylated cancer cell lines (HepG2) and two *Mieap*-methylated cancer cell lines (H1299 and U373MG) indicated that exogenous Mieap was localized to both cytoskeletal-like and granule-like regions of the cytoplasm (Figure S3A). Interestingly, the granular signals of Mieap were colocalized with the mitochondrial signals and those of lysosomes (Figure S3A). In addition, at the same time, the mitochondrial signals almost completely colocalized with those of lysosomes (Figure S3A). We did not observe any overlap of the endoplasmic reticulum (ER) and lysosomal signals or the Golgi and lysosomal signals in the cells (data not shown).

**Figure 3:**
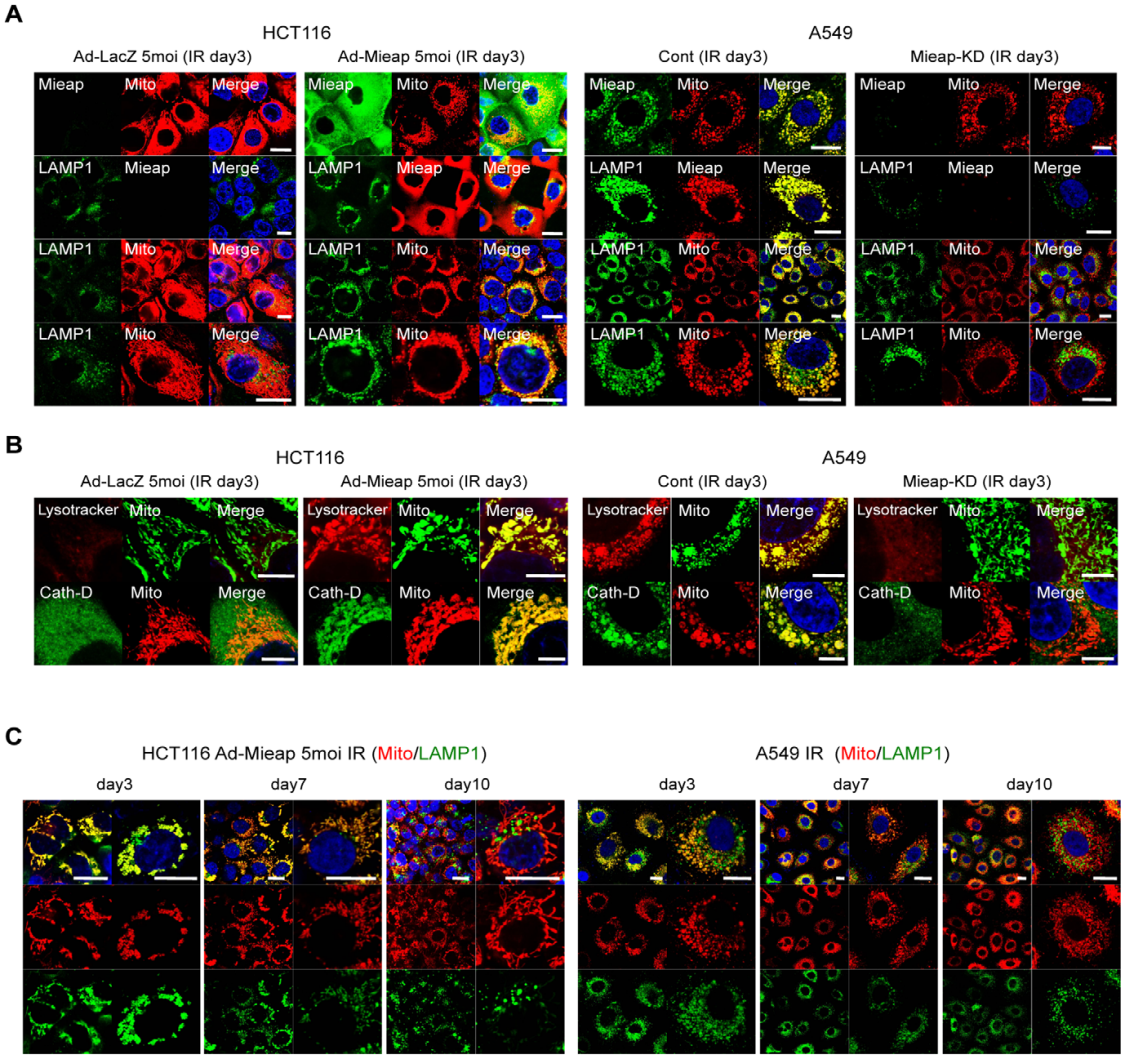
Mieap induces accumulation of lysosomes in mitochondria. (A) Mieap induces the colocalization of lysosomes with mitochondria. The Ad-LacZ and Ad-Mieap infected cells of HCT116, or the cont and Mieap-KD cells of A549 were subjected to IF experiment on day 3 after IR. Mieap protein was stained with polyclonal rabbit anti-Mieap antibody (Mieap: green or red). Lysosomes were stained with anti-LAMP1 antibody (LAMP1: green). Mitochondria were indicated by the DsRed-mito protein signal (Mito: red). Scale bar = 20 µm (B) Mieap-regulated lysosomes in mitochondria are active. The cells were subjected to IF experiment on day 3 after IR. Mitochondria were stained with AcGFP-mito (Mito: green) or DsRed-mito (Mito: red). Lysosomes were stained with Lysotracker-Red (Lysotracker: red) or anti-cathepsin D antibody (Cath-D: green). Scale bar = 10 µm (C) The phenomenon is reversible and continues for several days. Ad-Mieap-infected HCT116 cells or the cont cells of A549 were irradiated by γ ray, and subjected to IF experiment on day 3, 7 and 10 after IR. Lysosomes were stained with anti-LAMP1 antibody (LAMP1: green). Mitochondria were shown by the DsRed-mito protein signal (Mito: red). Scale bar = 20 µm.

Next, we confirmed the phenomenon on endogenous Mieap. In HCT116 *Mieap*-methylated cancer cells, we never observed the signal of the Mieap expression in Ad-LacZ infected HCT116 cells on day 3 after ionizing radiation (IR) (Figure 3A). Consistent with the lack of Mieap expression, we were also unable to detect accumulation of lysosomal signals in mitochondria in the irradiated cells (Figure 3A). However, once Mieap was expressed by infection with Ad-Mieap at an MOI of 5, accumulation of lysosomal signals in mitochondria was quickly induced in HCT116 cells (Figure 3A). The similar results were obtained in any *Mieap*-methylated cancer cells examined (Figure S3B and data not shown).

In contrast, in A549 *Mieap*-unmethylated cancer cells, the endogenous Mieap protein was intensively induced on day 3 after treatment of the cells with IR (Figure 3A). The Mieap expression pattern clearly overlapped with the mitochondrial and lysosomal signals (Figure 3A). In addition, colocalization of the lysosomal signals with the mitochondrial one was observed in the IR-treated A549 cells (Figure 3A). However, when Mieap expression was substantially inhibited in the *Mieap*-knockdown (KD) cells, the colocalization of the lysosomal and mitochondrial signals after IR was severely impaired (Figure 3A). The phenomenon was repeated after various mitochondrial stresses, including IR, Adriamycin treatment, or exposure to H_2_O_2_ (data not shown). These results suggest that endogenous Mieap may regulate the targeting of mitochondria by lysosomes in response to various mitochondrial stresses.

To address whether the observed lysosome-associated membrane protein 1 (LAMP1) signal is indicative of active lysosomes in the mitochondria, we carried out a Lysotracker experiment, which indicates the acidic status of lysosomes. As shown in Figure 3B, the strong red Lysotracker signals completely colocalized with the green mitochondrial signals, suggesting that the lysosomes in the mitochondria are active. We further confirmed that the Lysotracker signals are neutralized by the ammonium treatment, implying that the Lysotracker is specifically targeted to the acidic compartments (Figure S4). Moreover, we detected cathepsin D (Figure 3B) and cathepsin B (data not shown) in the mitochondria using anti-cathepsin D and cathepsin B antibodies. We also confirmed that the signals of LAMP1 and LAMP2 were clearly merged with those of Lysotracker and/or cathepsin D, implying that the signals of LAMP1 and LAMP2 are specifically indicating the localization of lysosomes (Figure S5).

We further examined whether the above phenomenon is irreversible or reversible, or how long the phenomenon continues. As shown in Figure 3C, in the irradiated A549 or Ad-Mieap-infected HCT116, lysosomes accumulated in almost all the mitochondria by day 3 after treatment and the accumulation of lysosomes in mitochondria reached its peak on day 5 (data not shown). However, on day 7, the phenomenon was weakened in some mitochondria. On day 10, in most of mitochondria, lysosomes and mitochondria were not merged, and the shape of lysosomes and mitochondria became normal (Figure 3C). These results suggest that the phenomenon is likely reversible, and that it takes several days to finish, indicating that the time course of the phenomenon is much longer than that of canonical autophagy. Canonical autophagy of mitochondria is a rapid process that usually finishes within a few hours [29]. In addition, we never observed loss of the mitochondrial signal during the process, whereas accumulation of lysosomes in mitochondria causes severe mitochondrial loss in canonical autophagy of mitochondria as reported previously [30], [31]. Therefore, we speculate that the phenomenon appears to be very different from canonical autophagy, but rather related to a repair process of the damaged mitochondria.

### Mieap-regulated lysosome in mitochondria is not related to canonical autophagy

To further explore the nature of the phenomenon, we used and compared two colorectal cancer cell lines, LS174T (*Mieap*-unmethylated) and HCT116 (*Mieap*-methylated). We confirmed that the colocalization of lysosomal signal with mitochondrial one was observed in the cont LS174T cells but was severely impaired in *Mieap*-KD LS174T cells on day 3 after IR (Figure S6).

**Figure 4:**
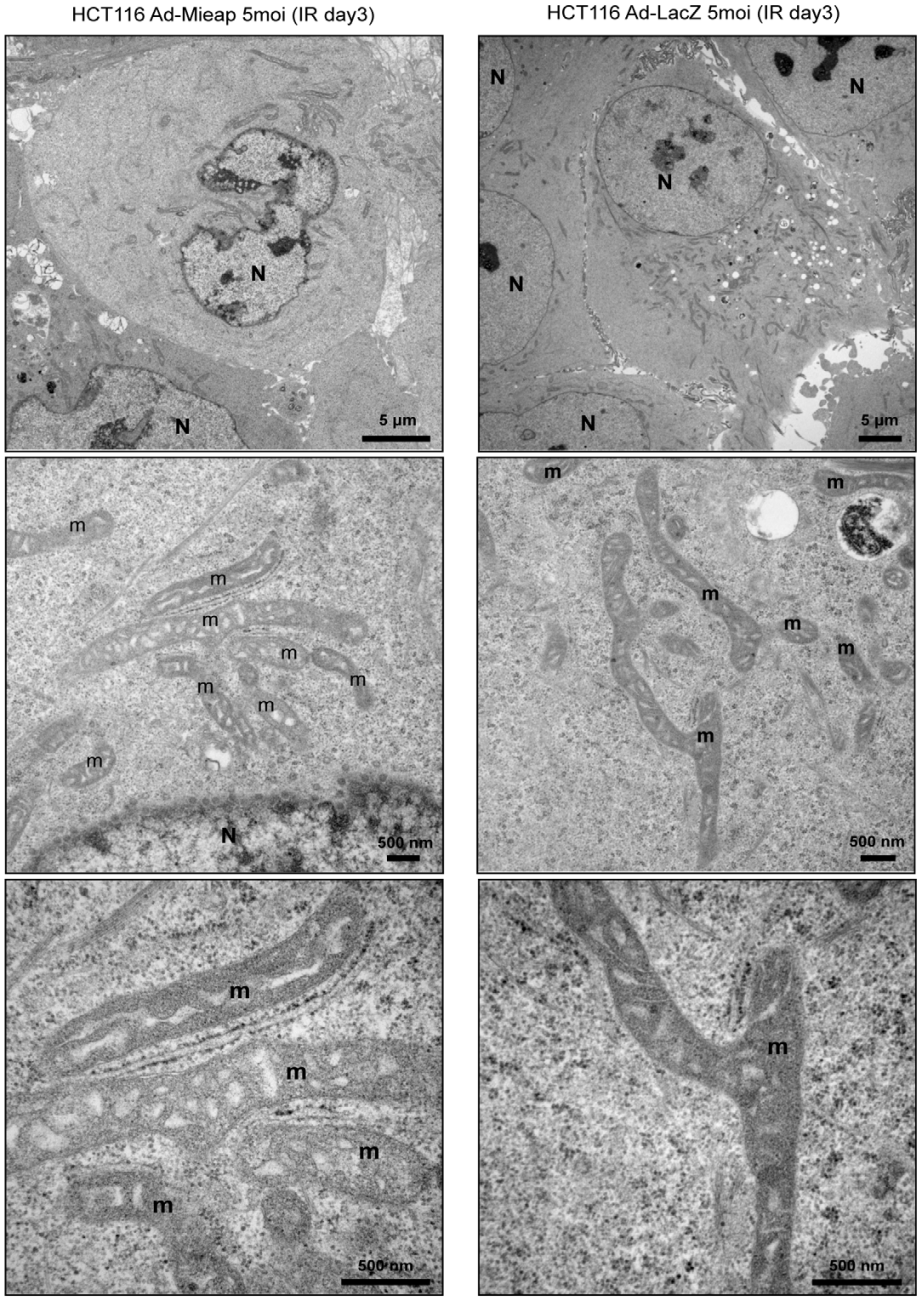
Electron Microscopic analysis of Ad-Mieap and Ad-LacZ infected HCT116 cells. The Ad-LacZ and Ad-Mieap infected cells of HCT116 were subjected to electron microscopic analysis on day 3 after IR. The representative images are shown. N: nucleus m: mitochondria Scale bar = 5 µm (upper) or 500 nm (middle and lower).

To assess whether Mieap-regulated lysosomes in mitochondria are related to canonical autophagy of mitochondria, which is mediated by double-membrane autophagosomes [10], [11], [29], [32], we conducted electron microscopy (EM) analysis on the Mieap^+^ cells (Ad-Mieap–infected HCT116, A549-cont and LS174T-control cells) and Mieap^−^ cells (Ad-LacZ–infected HCT116, A549-Mieap-KD and LS174–Mieap-KD cells) on day 3 after IR. Surprisingly, although lysosomal signals intensively accumulated in mitochondria as seen in the IF experiment (Figure 3A–3C), we could not detect any degraded structure of mitochondria in Mieap^+^ cells (Figure 4 and Figures S7 and S8). In addition, we could not detect any typical structure of lysosomes within mitochondria (Figure 4 and Figures S7 and S8). But, it is rather difficult to point out some morphological difference of mitochondria between the Mieap^+^ and Mieap^−^ cells (Figure 4 and Figures S7 and S8). In addition, autophagosomes or vacuoles both around and within the mitochondria of the Mieap^+^ cells were not observed (Figure 4 and Figures S7 and S8). However, we noticed that the cristae of the Mieap^+^ cells were rather clearer and more regular than that of the Mieap^−^ cells, implying that the morphology of the mitochondria of Mieap^+^ are more similar to that of healthy mitochondria, compared to those of Mieap^−^ cells (Figure 4 and Figures S7 and S8).

Since accumulation of the GFP-LC3 signal around mitochondrial outermembrane was reported in canonical autophagy of mitochondria [29], [30], [31], we examined the relationship between the LC3 and mitochondrial signals during the process of the phenomenon. However, we confirmed that the GFP-LC3 signal was not related to either Mieap-regulated lysosomes or mitochondria (Figure S9). On the basis of these findings, we concluded that the phenomenon induced by Mieap is completely different from canonical autophagy of mitochondria, and speculated that Mieap may induce atypical lysosome or lysosome-like organella within mitochondria.

### Mieap induces accumulation of lysosomal proteins within mitochondria without destroying the mitochondrial structure

**Figure 5:**
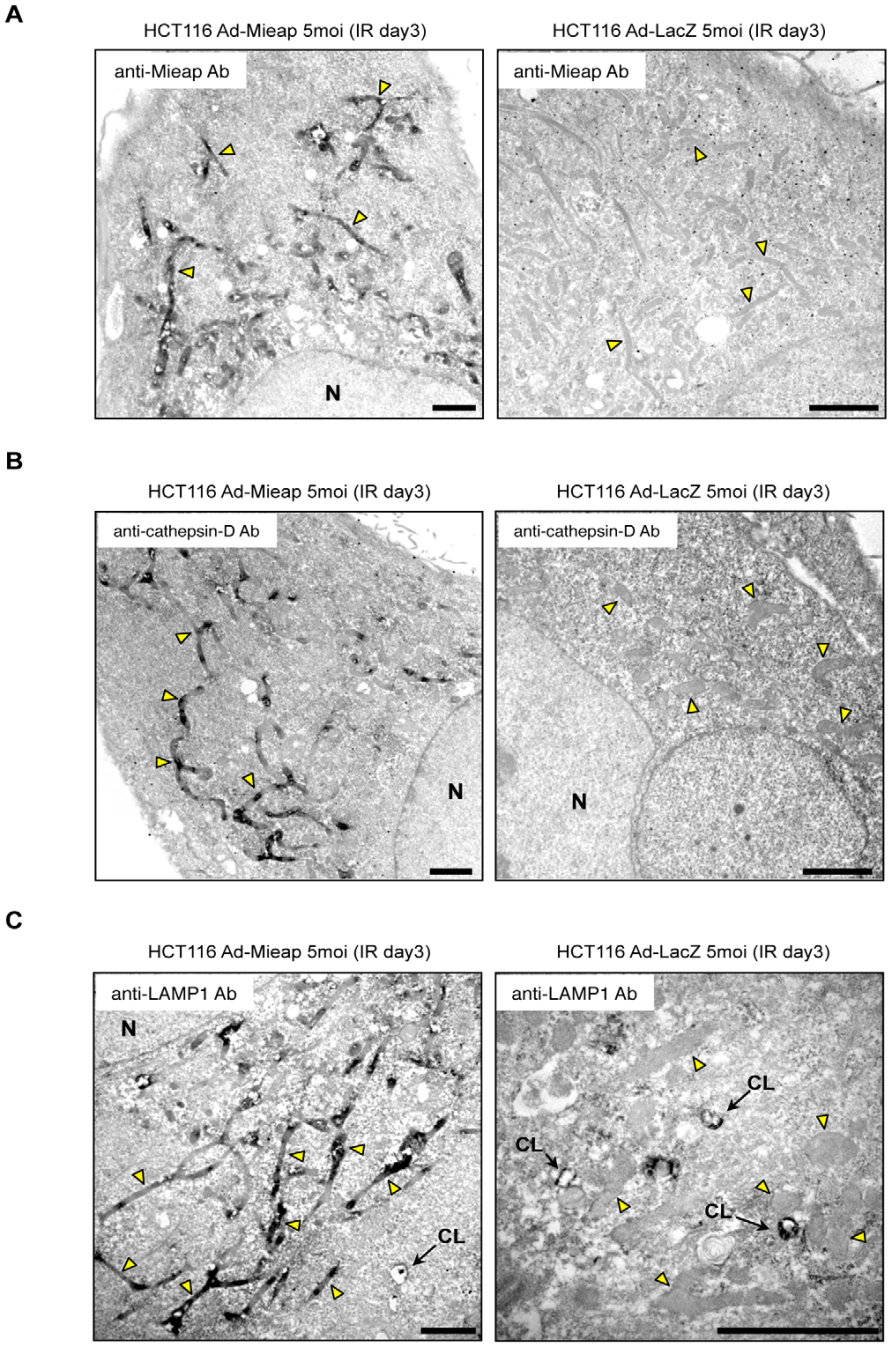
Pre-embedding immunoelectron microscopic analysis shows the presence of Mieap and lysosomal proteins within mitochondria. The Ad-Mieap and Ad-LacZ infected cells of HCT116 were subjected to pre-embedding imunoelectron microscopic analysis with DAB on day 3 after IR. The primary antibody against Mieap (A), cathepsin D (B), or LAMP1 (C) was used for the analysis. The representative images are shown. Arrowheads (yellow) indicate representative mitochondria. N: nucleus CL: cytoplasmic lysosome Scale bar = 2 µm.

To determine whether lysosomal proteins accumulate within mitochondria, we carried out pre-embedding immunoelectron microscopic analysis using diaminobenzidine tetrahydrochloride (DAB) and primary antibodies against Mieap, LAMP1, LAMP2, cathepsin D, or cathepsin B. Under the conditions shown in Figures 3 and 4, and Figures S7, S8, S9, all signals of Mieap (Figure 5A and Figure S10), cathepsin D (Figure 5B and Figure S11), LAMP1 (Figure 5C and Figure S12), LAMP2 (Figure S13), and cathepsin B (Figure S14) proteins were strongly detected as DAB products within the mitochondria of Ad-Mieap-infected HCT116 cells on day 3 after IR, whereas none of these protein signals could be detected within the mitochondria of Ad-LacZ-infected HCT116 cells (Figure 5 and Figures S10, S11, S12, S13, S14).

**Figure 6:**
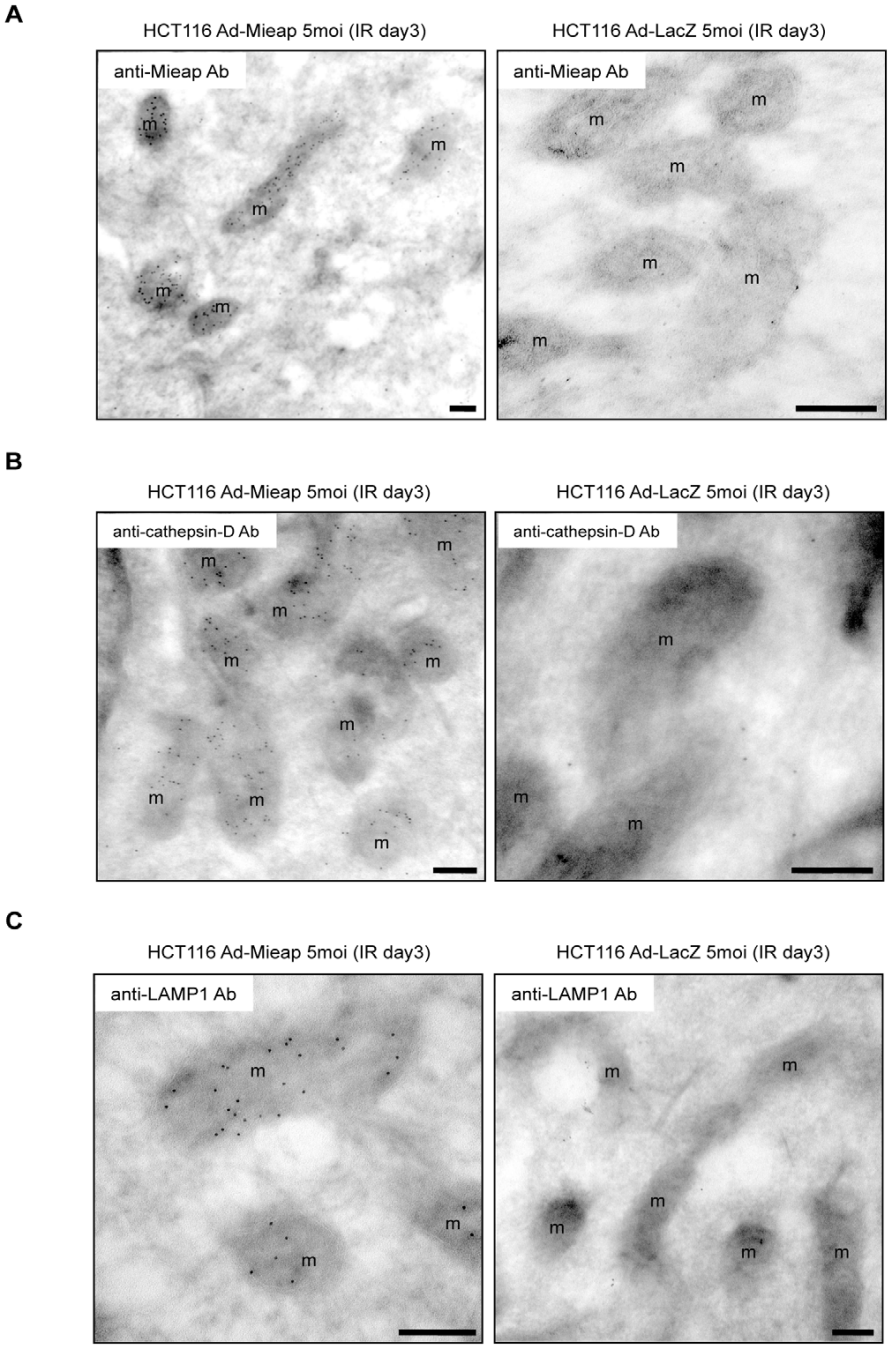
Post-embedding immunoelectron microscopic analysis shows the presence of Mieap and lysosomal proteins within mitochondria. The Ad-Mieap and Ad-LacZ infected cells of HCT116 were subjected to post-embedding imunoelectron microscopic analysis with gold particles on day 3 after IR. The primary antibody against Mieap (A), cathepsin D (B), or LAMP1 (C) was used for the analysis. The representative images are shown. m: mitochondria Scale bar = 200 nm.

To confirm the results of the pre-embedding method, we carried out post-embedding immunoelectron microscopic analysis using gold particles and primary antibodies against Mieap, LAMP1, or cathepsin D. Consistent with the result of the pre-embedding method, gold particle clusters indicating Mieap, cathepsin D, or LAMP1 labeling were observed within the mitochondria of Ad-Mieap-infected HCT116 cells on day 3 after IR, whereas no clusters were detected in the mitochondria of Ad-LacZ-infected cells (Figure 6A–6C). Furthermore, at high magnification, all Mieap, cathepsin D, and LAMP1 proteins may have been predominantly localized to the mitochondrial matrix (Figures S15, S16).

**Figure 7:**
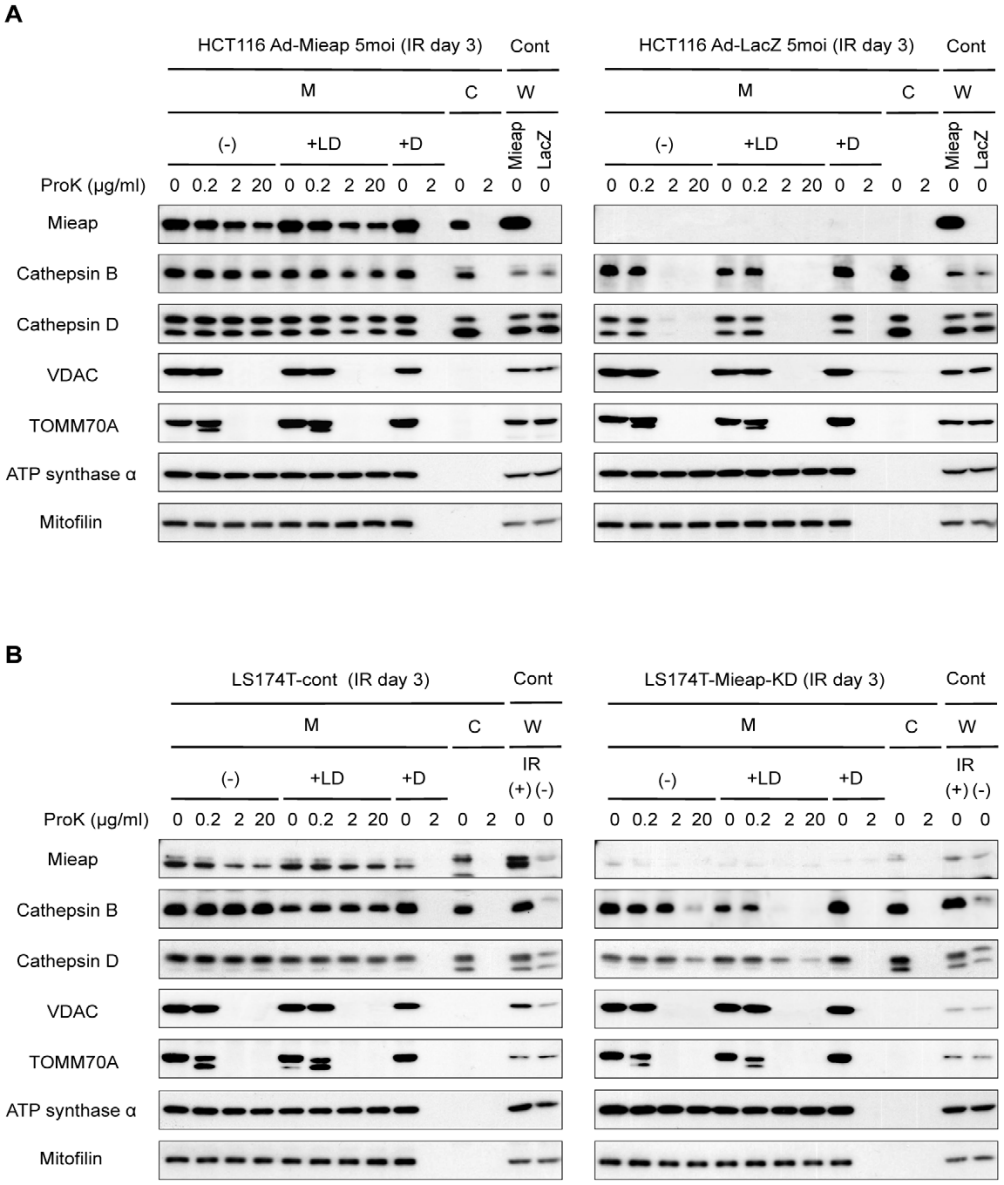
Proteinase K protection assay shows the presence of Mieap and lysosomal proteins within mitochondria. The mitochondria were fractionated from the Ad-LacZ and Ad-Mieap infected cells of HCT116 (A), or the cont and Mieap-KD cells of A549 (B) on day 3 after IR. The mitochondrial fraction was subjected to proteinase K protection assay. (-): no treatment, +LD: detergent treatment for lysosomal membrane disruption (100 µM Sphingosine), +D: detergent treatment for mitochondrial membrane disruption (1% Triton X-100), Mieap: Ad-Mieap infected HCT116 cells, LacZ: Ad-LacZ infected HCT116 cells, IR: ionizing radiation, M: mitochondrial fraction, C: cytoplasmic fraction, W: whole cell lysate, Cont: positive control for western blotting.

To further confirm the presence of lysosomal proteins within mitochondria, we carried out proteinase K protection assay with the fractionated mitochondria of Mieap^+^ cells (Ad-Mieap-infected HCT116 cells and LS174T control cells) and Mieap^−^ cells (Ad-LacZ-infected HCT116 cells and LS174 Mieap-KD cells) on day 3 after IR. The Mieap protein was detected in the mitochondrial fraction of the Ad-Mieap-infected HCT116 cells, whereas it was not detected in that of Ad-LacZ-infected HCT116 cells (Figure 7A). Two mitochondrial outermembrane proteins—voltage-dependent anion-selective channel protein 1 (VDAC/Porin) and translocase of outer mitochondrial membrane 70 homolog A (TOMM70A)—were degraded by addition of 0.2 or 2µg/ml proteinase K, whereas the Mieap protein in the mitochondrial fraction of the Ad-Mieap-infected HCT116 cells was not degraded by addition of even 20 µg/ml proteinase K (Figure 7A). This result indicates that the Mieap protein in the Ad-Mieap-infected HCT116 cells is localized within mitochondria. We further confirmed the presence of lysosomal proteins within mitochondria in this assay. Lysosomes are usually co-fractionated in the mitochondrial fraction by the method used in the present study (see Materials and Methods). Therefore, we treated the lysosome in the mitochondrial fraction with 100 µM sphingosine in order to disrupt the lysosomal membrane. Two lysosomal enzyme proteins—cathepsin D and cathepsin B—in the mitochondrial fraction of the Ad-Mieap-infected HCT116 cells were also resistant to proteinase K digestion even after destruction of the lysosomal membrane, whereas those in the mitochondrial fraction of the Ad-LacZ-infected HCT116 cells were degraded by addition of 2 µg/ml proteinase K, regardless of the presence or absence of sphingosine (Figure 7A). This result clearly suggests that lysosomal proteins are present within mitochondria. Similar results were obtained in the experiment on endogenous Mieap in LS174T cells (Figure 7B).

We further showed that anti-cathepsin D and anti-cathepsin B antibodies specifically detected and indicated the predicted sizes of cathepsin D and cathepsin B proteins by western blotting (Figure S17). Moreover, the results also indicated that the molecular weights of the intramitochondrial proteins of cathepsin D and cathepsin B (M: lanes 1–10) are the same as those of the cytoplasmic proteins of cathepsin D and cathepsin B (C: lanes 11–12), implying that the intramitochondrial cathpsin D and cathepsin B are not synthesized within mitochondria, but the cytoplasmic proteins are translocated into mitochondria (Figure S17).

All these results strongly indicated that Mieap and lysosomal proteins are localized within mitochondria. Therefore, we designated this phenomenon as Mieap-induced accumulation of lysosome-like organelles within mitochondria (MALM).

### Mieap-induced intramitochondrial lysosome-like organella is involved in eliminating oxidized mitochondrial proteins

**Figure 8:**
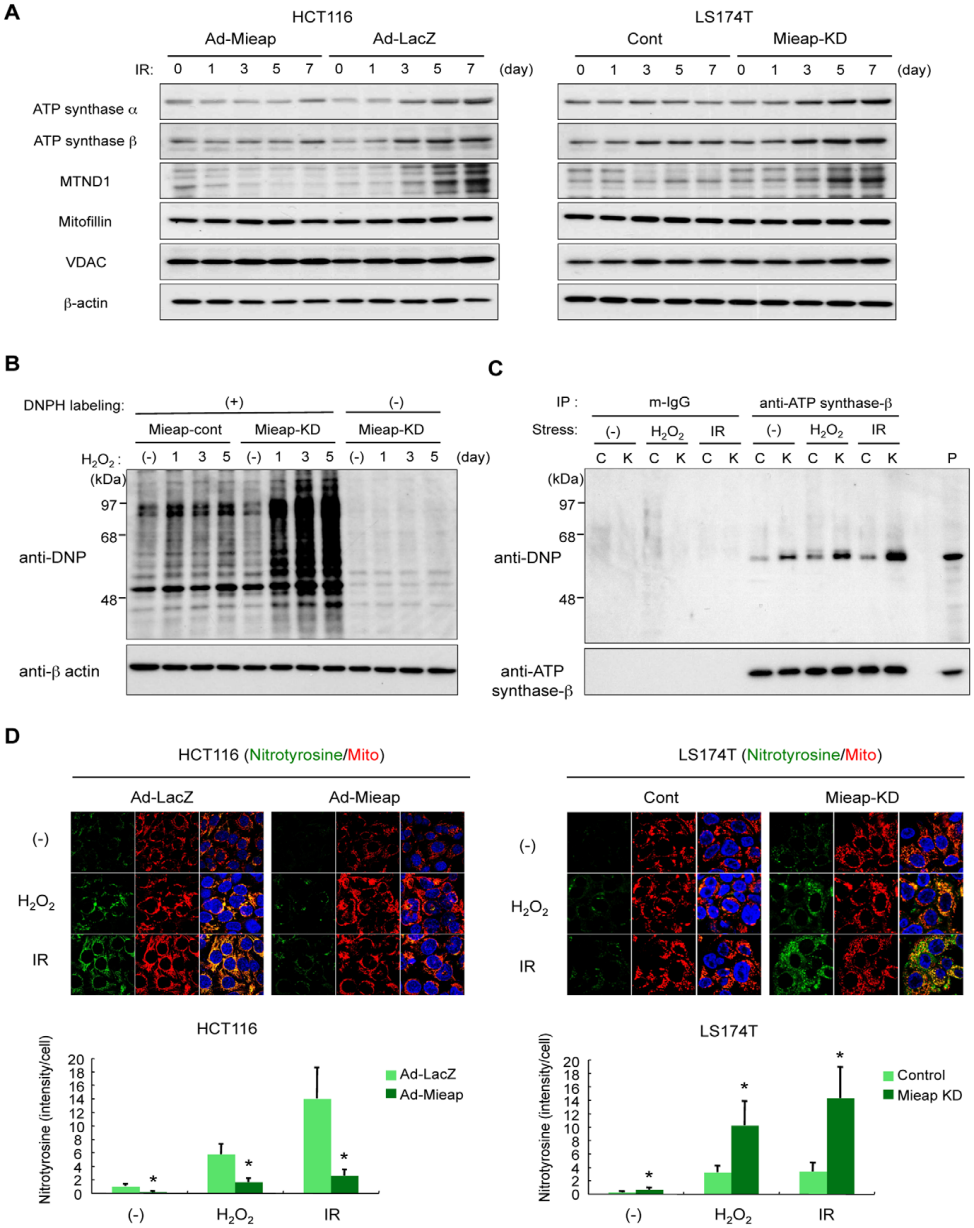
MALM is involved in elimination of mitochondrial oxidized proteins. (A) Expression levels of endogenous mitochondrial proteins. The Ad-Mieap- or Ad-LacZ-infected HCT116 cells, or the LS174T cont and Mieap-KD cells were irradiated by γ ray, and the cells were subjected to western blot analysis at the indicated times on the indicated proteins. (B) Western blot analysis of the oxidized proteins. The total cell lysates isolated at the indicated times from the H_2_O_2_-treated cont and Mieap-KD cells of LS174T were labeled by DNPH, and then subjected to western blot analysis with anti-DNP antibody in order to detect the carbonyl-oxidized proteins. β-actin was used as a loading control (A) (B). (C) Western blot analysis of the oxidized ATP synthase beta-subunit protein. ATP synthase beta-subunit protein was specifically immunoprecipitated with mouse monoclonal anti-ATP synthase beta-subunit antibody from the cell lysates isolated from the non-treated (-), H_2_O_2_-treated (H_2_O_2_), or γ-irradiated (IR) cont (C) and Mieap-KD (K) cells of LS174T, and then the precipitated proteins in each group were subjected to DNPH labeling and the following western blot analysis with anti-DNP antibody. Total ATP synthase beta-subunit protein in the stripped membrane was detected with rabbit polyclonal anti-ATP synthase beta-subunit antibody as the loading control. The band of ATP synthase beta-subunit protein was shown as the positive control of the size (P). (D) IF analysis of the oxidized proteins. The Ad-Mieap- or Ad-LacZ-infected HCT116 cells, or the LS174T cont and Mieap-KD cells were irradiated by γ ray, treated with H_2_O_2_, or not treated, and 3 days after the treatment, the IF experiment was carried out with anti-nitrotyrosine antibody (Nitrotyrosine) in order to detect the nitrotyrosine-oxidized proteins. Mitochondria were indicated by the DsRed-mito protein signal (Mito). The representative images were shown (upper panel). Quantitative analysis of nitrotyrosine intensity was carried out in 300–400 cells. Average intensities of the nitrotyrosin-oxidized proteins per cell are shown with error bars indicating 1 standard deviation (SD; lower panel). p<0.01 (*) was considered statistically significant.

Since MALM is not related to canonical autophagy of mitochondria, we speculated that MALM might be involved in degradation of the mitochondrial proteins. The expression levels of endogenous mitochondrial proteins were assessed in the Mieap^+^ cells (Ad-Mieap–infected HCT116 and LS174T-control cells) and Mieap^−^ cells (Ad-LacZ–infected HCT116 and LS174–Mieap-KD cells). Interestingly, F1F0-ATP synthase alpha-subunit (ATP synthase α), beta-subunit (ATP synthase β), and mitochondrial DNA-encoded NADH dehydrogenase subunit 1 (MTND1), which are located in mitochondrial matrix and innermembrane and are critical mediators for oxidative phosphorylation and energy production, slightly accumulated after IR in the Mieap^−^ cells (Figure 8A). However, no differences in mitofilin (intermembrane space protein) and VDAC (outermembrane protein) between the Mieap^+^ and Mieap^−^ cells were observed (Figure 8A). These results suggest that MALM may specifically degrade some mitochondrial proteins in mitochondrial matrix and innermembrane proteins.

Since mitochondria are the main source of ROS generation in cells, the mitochondrial proteins are often oxidized and damaged by ROS [7], [8], [12]. Thus, we speculated that the Mieap-regulated lysosomes might specifically target and degrade the oxidized proteins in mitochondria. To confirm this hypothesis, we analyzed the oxidized protein.

First, we examined the oxidative modification of carbonyl groups in protein side chains. As shown in Figure 8B, oxidized proteins markedly accumulated in LS174T–*Mieap*-KD cells following treatment with H_2_O_2_ in a time-dependent manner, whereas no change in LS174T-control cells was observed. Furthermore, one of the oxidized proteins was identified as the F1F0-ATP synthas β-subunit (Figure 8C), which plays an essential role in mitochondrial ATP production. Interestingly, prior to the application of any stress, the carbonylated form of the F1F0-ATP synthase β-subunit was already increased in the Mieap^−^ cells (Figure 8C), suggesting that MALM functions under normal basal conditions to eliminate ROS-damaged proteins in mitochondria.

Next, we examined the other oxidative modification of nitrotyrosine using anti-nitrotyrosine antibody that allows for IF analysis of the oxidized proteins. As shown in Figure 8D, both the IR and H_2_O_2_ treatment dramatically induced the accumulation of oxidative proteins in the mitochondria, and in part, in the cytoplasm outside of the mitochondria in the Mieap**^−^** cells (Ad-LacZ infected HCT116 and LS174T-Mieap-KD). In contrast, the Mieap**^+^** cells (Ad-Mieap-infected HCT116 and LS174T-cont) revealed much smaller changes. Consistent with the results of western blot analysis on carbonyl groups modification, even without any stresses, the Mieap**^−^** cells already revealed accumulation of nitrotyrosine-oxidized proteins in mitochondria. These results further support the role of Mieap in the maintenance of mitochondrial integrity under normal condition (Figure 8D). Collectively, the present results suggest that, in order to maintain healthy mitochondria, Mieap-induced intramitochondrial lysosome-like organella plays an important role in eliminating the oxidized mitochondrial proteins.

### Mieap repairs unhealthy mitochondria

Oxidized proteins have been reported to lose their normal activity [14], [33]. Therefore, it is possible that accumulation of the oxidized proteins in mitochondria may impair the function of the mitochondria, leading to the accumulation of dysfunctional and unhealthy mitochondria in the Mieap^−^ cells. The term “unhealthy mitochondria” generally refers to mitochondria with dysfunctional oxidative phosphorylation, which is manifested by reduced ATP synthesis and the excess generation of ROS [7]. In addition, the oxidized form of the F1F0-ATP synthase β-subunit was actually accumulated in the Mieap^−^ cells (Figure 8C). Therefore, we hypothesized that the mitochondria in the Mieap^−^ cells might show reduced ATP synthesis and high levels of ROS.

**Figure 9:**
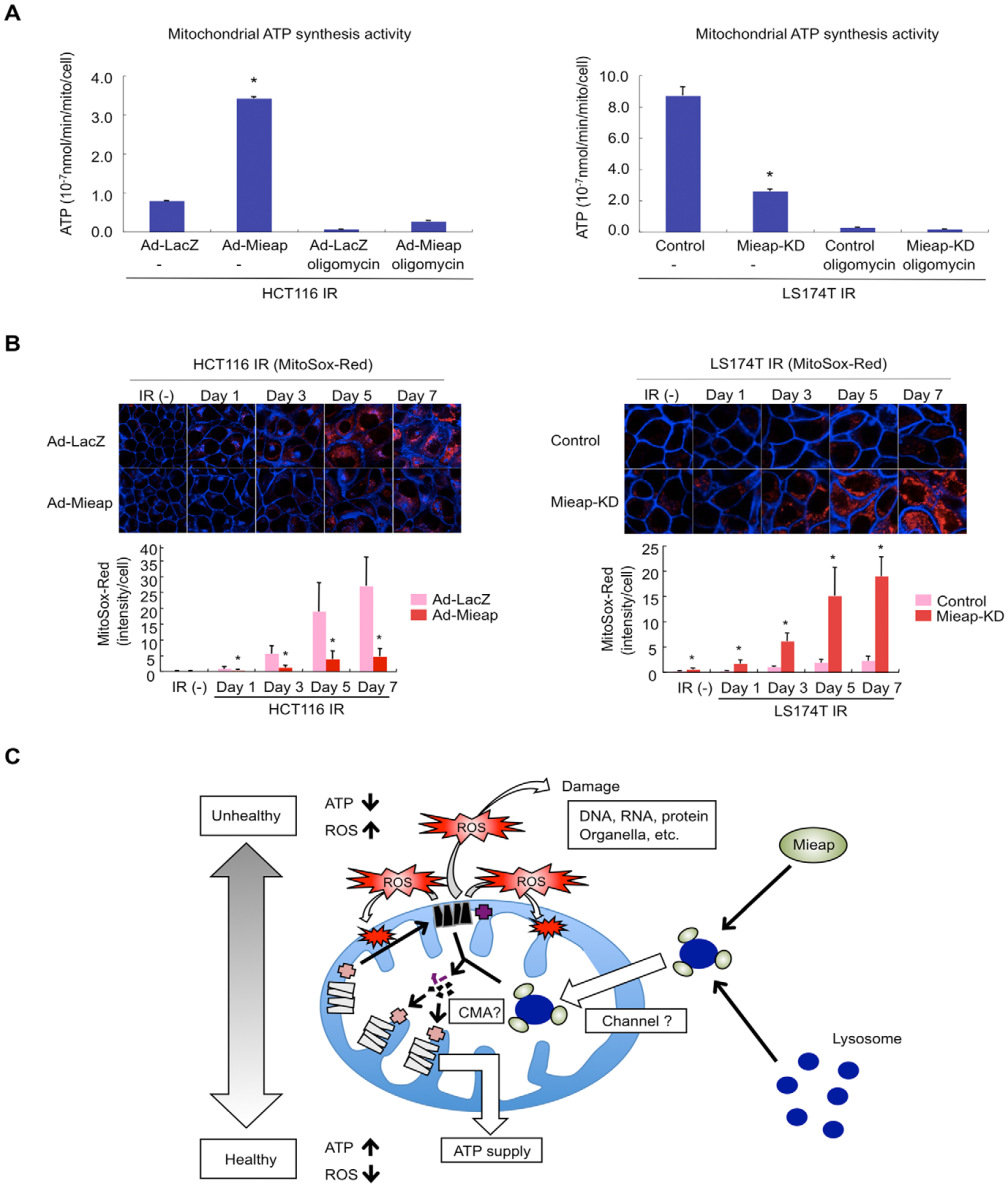
Mieap repairs unhealthy mitochondria. (A) ATP synthesis activity by the mitochondria. The Ad-Mieap- or Ad-LacZ-infected HCT116 cells, or the LS174T cont and Mieap-KD cells were subjected to ATP synthesis assay on day 7 after IR. Oligomycin, an inihibitor of mitochondrial oxidative phosphorylation, was used in the assay in order to detect non-mitochondrial ATP synthesis activity. The assay of each group was independently carried out three times. The average activities of ATP synthesis are shown with error bars indicating 1 SD. p<0.01 (*) was considered statistically significant. (B) Mitochondrial ROS level. The cells were γ ray irradiated, and the ROS generated by mitochondria in the cells was analyzed by MitoSox-Red without IR or at the indicated times after IR. The representative images are shown (upper panel). Quantitative analysis of ROS was carried out in 300–400 cells (lower panel). Average intensities of ROS per cell are shown with error bars indicating 1 SD. p<0.01 (*) was considered statistically significant. (C) Hypothetical model for the Mieap-regulated mitochondrial quality control.

To investigate whether the mitochondria in the Mieap-deficient cells are dysfunctional, the ATP synthesis activity of the mitochondria in the Mieap^+^ (Ad-Mieap-infected HCT116 and LS174T-cont) and Mieap^−^ (Ad-LacZ infected HCT116 and LS174T-Mieap-KD) cells was assessed as previously described [34]. Interestingly, the ATP synthesis activity of the mitochondria was significantly impaired in the Mieap^−^ cells even prior to the induction of IR-mediated stress (data not shown). Moreover, the ATP synthesis activity of the mitochondria in the Mieap^−^ cells was severely decreased following IR-treatment compared to the Mieap^+^ cells (Figure 9A).

Further examination of the ROS generated by mitochondria in the Mieap-deficient cells, using the mitochondrial ROS indicator MitoSOX-Red (red), indicated that the mitochondrial ROS level in both Mieap^−^ cell lines dramatically increased after IR stress in a time-dependent manner, whereas the Mieap^+^ cells showed only a slight increase (Figure 9B). Additional experiments with another mitochondrial ROS indicator, dihydrorhodamine123 (DHR123) (green), further verified these findings (data not shown). Taken together, these results suggest that Mieap prevents the accumulation of unhealthy and dysfunctional mitochondria by eliminating the oxidized and damaged mitochondrial proteins, including ATP synthase β-subunit, thereby maintaining mitochondrial function and inhibiting the generation of mitochondrial ROS.

## Discussion

Canonical autophagy of mitochondria, known as “mitophagy,” is thought to play a critical role in mitochondrial quality control [10], [11]. To date, studies on yeast have provided most of the information on mitophagy. Several yeast studies have suggested that mitochondrial autophagy is mediated by macroautophagy [11], [35], and studies on mammalian cells have supported this finding [29], [36]. In macroautophagy, double-membraned autophagosomes engulf proteins and organelles along with a portion of the cytoplasm [32]. Therefore, the process of macroautophagy is generally considered non-specific to the target proteins and organelles. However, it has been suggested that some type of mitophagy can be mediated by the selective macroautophagy of mitochondria. For example, NIX has been shown to play an essential role in the selective mitochondrial macroautophagy observed during the maturation of erythroid cells [37], [38]. In cervical cancer and neuroblastoma cells, parkin and PINK1 have also been shown to play a critical role in the selective macroautophagy of mitochondria [30], [31]. In all these mechanisms, double-membraned autophagosomes are essential for the degradation of the entire structure of mitochondrion. In addition, the process is very rapid and is completed within a few hours [29], [36]. However, in the present study, MALM continued for several days, and autophagosomes characterized by the double-membraned structure on electron microscopy (EM) and the dot signal of GFP-LC3 on immunofluorescence (IF) analysis were not related to MALM at all. Moreover, we observed neither the destruction of the mitochondrial structure on EM nor the loss of mitochondrial signal on IF analysis; these findings are usually detected in canonical autophagy of mitochondria. On the basis of these facts, we conclude that MALM is completely different from degradation of the entire mitochondrion, which is also termed mitophagy.

From the morphological viewpoint, a typical lysosomal structure in the cytoplasm is a membrane-surrounded vesicle, whose size varies from less than 1 µm (∼10 nm) to several microns and whose content reveals a heterogeneous and variable pattern, which occasionally contains other organelles for digestion [16], [39]. On EM, we were unable to detect this type of lysosomal structure within mitochondria. However, pre-embedding immunoelectron microscopy with DAB clearly showed that Mieap and lysosomal proteins, including LAMP1, LAMP2, cathepsin D, and cathepsin B, are located within mitochondria. In addition, post-embedding immunoelectron microscopy with gold particles also showed the presence of the Mieap, cathepsin D, and LAMP1 proteins within mitochondria. Furthermore, the results of proteinase K protection assay with the fractionated mitochondria indicated that the Mieap, cathepsin B, and cathepsin D proteins are present within mitochondria. On the basis of these facts, we concluded that atypical lysosomes or lysosome-like organelles are localized within mitochondria, without destroying the mitochondrial structure. Therefore, we speculate that the morphological structure of the Mieap-induced intramitochondrial lysosome-like organella is different from the typical lysosomal structure.

The regular structure of lysosomes is difficult to describe. In terms of morphology, lysosomes are less clearly defined than other subcellular organelles [16], [39]. The lysosomal structure is variable and depends on the cell type and the actual conditions [16], [39]. Even when lysosomes are visible on EM, their size varies from less than 1 µm (∼10 nm) to several microns [16], [39]. In particular, the minimum size of lysosomes is unknown. These findings indicate that the shape and appearance of lysosomes have not been clearly defined thus far. Mitochondria contain a number of membrane structures because of the presence of mitochondrial cristae and the inner membrane. Therefore, it appears to be difficult to determine the lysosomal structure within mitochondria by distinguishing the lysosomal membrane from the mitochondrial membrane. In particular, when a lysosome is involved in a specific degradation of proteins, such as chaperon-mediated autophagy (CMA) [19], it is much more difficult to determine the lysosomal structure because the lysosome does not contain any degraded contents of some organelle structures. Currently, it is likely that only the immunocytochemical or biochemical analysis carried out in this study can be used to detect the presence of intramitochondrial lysosome-like organella.

The presence of intramitochondrial lysosome-like organella raises many questions. One of the most important questions is how the organelles occur within mitochondria. We hypothesize that the presence of intramitochondrial lysosome-like organella can be explained via two possible mechanisms. First, cytoplasmic lysosomes or lysosome-like organelles are translocated into mitochondria without destroying the mitochondrial outer and inner membranes. Second, lysosomes or lysosome-like organelles are generated within mitochondria. We assume that the former mechanism is more plausible than the latter because the lysosomal compartment consists of at least more than 50 proteins, as mentioned. Because mitochondrial DNA does not encode lysosomal proteins, all the components of lysosomes must be imported into mitochondria before assembly. However, none of the lysosomal components have mitochondria-targeting signal sequences, implying that the proteins cannot be delivered into mitochondria. In addition, the organella is surrounded by a single membrane structure. It appears almost impossible to supply or generate the lysosomal membrane structure within mitochondria. Moreover, as shown in Figure S17, the molecular weights of the intramitochondrial proteins of cathepsin D and cathepsin B in MALM are the same as those of the cytoplasmic proteins of cathepsin D and cathepsin B, implying that the intramitochondrial cathpsin D and cathepsin B are not synthesized within mitochondria, but the cytoplasmic proteins are translocated into mitochondria. When cathepsin D and cathepsin B proteins are synthesized in cytoplasm, the proteins are targeted to lysosomes through ER-Golgi trafficking pathway. During the process, cathepsin D and cathepsin B are glycosylated. If the intramitochondrial cathepsin D and cathepsin B are synthesized within mitochondria, the intramitochondrial proteins are not glycosylated, whose molecular-weights are different from those of the cytoplasmic proteins. Therefore, taken together, we think that the latter mechanism is unfeasible and very inefficient. We speculate that cytoplasmic lysosome-like organelles are translocated into mitochondria.

Even if our hypothesis is true, it remains to be determined how lysosome-like organelles enter mitochondria without breaking down the mitochondrial structure. Several channels of the mitochondrial outer and inner membranes, which regulate the transport of mitochondrial proteins [40] or the release of cytochrome *c* from the mitochondrial intermembrane space during apoptosis [41], have been identified. None of these channels appear to explain the mechanism underlying MALM because the protein transport machinery can allow the specific transport of only individual proteins [40], and the pore of the apoptosis machinery such as VDAC seems to open a hole in only the mitochondrial outer membrane in order to release cytochrome *c* [41]. However, we speculate that a channel such as the mitochondrial permeability transition pore (MPTP) may mediate the translocation of lysosome-like organelles into mitochondria. MPTP is thought to be a nonspecific channel that spans the mitochondrial outer and inner membranes and creates a large hole between the cytoplasm and the mitochondrial matrix, leading to equilibration of H^+^ across the inner membrane and mitochondrial swelling due to water influx [42]. Therefore, MPTP has been suggested to play a role in atypical cell death such as necrosis. However, the physiological role of MPTP remains unknown [42]. Because MPTP itself is permeable to solutes with a size of up to 1.5 kDa, the size of the pore seems to be too small to allow the translocation of lysosomes or lysosome-like organelles. Therefore, we speculate that an unidentified MPTP-like channel may open a large hole from the mitochondrial outer membrane to the inner membrane under the regulation of Mieap and enable translocation of lysosome-like organelles from the cytoplasm into mitochondria (Figure 9C). Further investigation is required to examine this hypothesis.

Because mitochondria produce the majority of ROS within the cell, proteins in the mitochondria are the primary targets for mitochondrial ROS and oxidative damage [8], [12]. The oxidatively damaged proteins are thought to lose their normal function, which can lead to the loss of normal mitochondrial function [14], [33]. Thus, the mechanism of degradation of the oxidized proteins in mitochondria appears to be important for the maintenance of mitochondrial quality. Currently, this quality control mechanism of mitochondrial proteins has not been fully characterized and only some components of the mechanism of protein degradation within mitochondria have been identified. For example, several proteases can play a critical role in this regulation of protein degradation within the mitochondria [13]. Among them, the LON protease is an important factor in the degradation of oxidized proteins in the mitochondrial matrix, including Aconitase [14], [43]. In addition to LON, AAA proteases, including i-AAA and m-AAA, are also thought to be involved in the degradation of mitochondrial inner-membrane proteins [44]. Thus, these proteases play a critical role in mitochondrial protein degradation in order to maintain the quality of the mitochondria. If so, what is the role of MALM? Since the protease activities of AAA and LON primarily depend on ATP, they can function when ATP concentration is sufficient [13]. Therefore, when the cell experiences various stressful events leading to mitochondrial damage, mitochondrial ATP production may be reduced. Such conditions may result in the impairment of LON and i- or m-AAA protease function. Therefore, it is possible that the MALM system in mitochondria is a back-up system for stressful conditions, which functions to eliminate the damaged proteins within mitochondria to accelerate the repair process of unhealthy mitochondria.

In the present study, we have shown that intramitochondrial lysosome-like organella are involved in the degradation of oxidized proteins in mitochondria. The important question is how the organelles can degrade the oxidized proteins within mitochondria. We speculate that one existing mechanism may be the model mechanism underlying this function. CMA specifically degrades the oxidized proteins in cytoplasm and is one of the three major pathways in autophagy; however, it is considered an atypical function of lysosomes [19]. In this mechanism, the lysosomes can specifically uptake the oxidized proteins containing the consensus motif via the interplay of LAMP2A and heat shock protein 70 (HSP70) [19]. If the entire structure of the lysosome or lysosome-like organelles can enter and exist within the mitochondrion, MALM may function as the intramitochondrial CMA-like mechanism in order to eliminate and degrade oxidized proteins (Figure 9C).

p53 has been reported to regulate aerobic respiration by the transcriptional activation of p53 targets that are involved in the mitochondrial electron transport chain, such as SCO2 (24). In addition, p53R2, a p53 target, has been shown to regulate mitochondrial DNA synthesis [45]–[47]. These findings suggest that the inactivation of *p53* in human cancers leads to an impairment of aerobic respiration and mitochondrial DNA synthesis via downregulation of SCO2 and p53R2, respectively. In addition, *p53* mutations cause the accumulation of dysfunctional mitochondrial proteins via downregulation of *Mieap*. Moreover, *Mieap* expression is directly inactivated by methylation of its promoter. This causes the accumulation of unhealthy mitochondria, suggesting that these organelles in cancer cells tend to become dysfunctional. This might explain, in part, why cancer cells preferentially utilize aerobic glycolysis, as observed by Warburg [20]. Therefore, we suggest that cancer cells have a predisposition to accumulate unhealthy and dysfunctional mitochondria.

Although there are many important questions that require further study, the discovery of this unusual and important mechanism in the cell may lead to a greater understanding of the underlying mechanisms involved in various phenomena and diseases that may be a result of defective mitochondrial quality control. Moreover, our discovery raises a number of important questions regarding the well-established concepts of cell biology.

## Materials and Methods

### Cell lines

The following human cancer cell lines were purchased from the American Type Culture Collection: LS174T, HCT116, HT29 and Lovo (colorectal adenocarcinoma); HepG2 (hepatoblastoma); A549 and H1299 (lung cancer); MCF7 and T47D (mammary carcinoma); SKN-AS (neuroblastoma); U87MG, U138MG, U373MG and T98G (glioblastoma); HeLa (cervical cancer); SaOS2 (osteosarcoma); and Tera2 (malignant embryonal carcinoma). The TERT-immortalized normal cell line HFF2 (human fibroblast cell) was provided by T. Kiyono (National Cancer Centre Research Institute) and D.A. Galloway (Fred Hutchinson Cancer Research Centre). The LC176 cell line (lung cancer) was a gift from T. Takahashi (Aichi Cancer Centre Research Institute). Cells were cultured under the conditions recommended by their depositors.

### Semi-quantitative RT-PCR analysis

The RT-PCR exponential phase was determined on 18–28 cycles, to allow semi-quantitative comparisons among complementary DNAs (cDNAs) developed from identical reactions. Each PCR regimen involved an initial denaturation step at 94°C for 5 min followed by 18 cycles (*β2-MG*), 19 cycles (*p21/WAF1*), 24 cycles (*Mieap*), 27 cycles (*ATG5* and *ATG12*) or 28 cycles (*BECN1* and *DRAM*) at 55°C for 30 s, and at 72°C for 30 s on a GeneAmp PCR system 9700 (Applied Biosystems). Primer sequences were, for *β2-MG*: forward, 5′-TAGCTGTGCTCGCGCTACT-3′ and reverse, 5′- GCGCTACTCTCTCTTCTG-3′; for *p21/WAF1*: forward, 5′- TTGGCCTGCCCAAGCTCTA-3′ and reverse, 5′-TCCTCTTGGAGAAGATCAGC-3′; for *Mieap*: forward, 5′-CTTTGCAATGCAGGCCTTAGA-3′ and reverse, 5′-CCGACTTCGAGACATCGTG-3′; for *ATG5*: forward, 5′- CTAAGGATGCAATTGAAGCTC-3′ and reverse, 5′-AGGAGATCTCCTAGTGTGTG-3′; for *ATG12*: forward, 5′-TCTGTGTTGCAGCTTCCTAC-3′ and reverse, 5′-CCATCACTGCCAAAACACTC-3′; for *BECN1*: forward, 5′-TGCTCTGGCCAATAAGATGG-3′ and reverse, 5′- CCTGTGTCTTCAATCTTGCC-3′; for *DRAM*: forward, 5′-TTAGTGCTTGGATTGGTGGG and reverse, 5′-CCACTGCCATTCACAGATC-3′.

### ChIP and gene reporter assays

The experiments were performed as described previously [27]. The 300-base pair (bp) fragment or the 358-bp fragment, including the potential p53-binding sites, BS1 or BS2, of *Mieap*, which were amplified with the same primers as used in the ChIP assays, were cloned into the pGL3 promoter vector (Promega). Primers sequences were, for BS1: forward, 5′-GAAGAAGTACAACGTGAGTC-3′ and reverse, 5′-GCGTCCTTAGTTACCACTGA-3′; for BS2: forward, 5′-TCTGTGCTTTGGAGACAGTAT-3′ and reverse, 5′-ATCTCATGAGCAGAGTCTTAGC-3′.

### MSP and bisulphite sequencing

MSP, bisulphite sequencing and 2′-deoxy-5-azacytidine (5-Aza-dC) treatment were performed as described previously [48]. The PCR reaction for MSP was carried out using the following conditions: 1 cycle at 94°C for 5 min, 30 cycles at 94°C for 30 s, 59°C for 30 s, and 72°C for 1 min, and a final extension step at 72°C for 7 min. Primers sequences were, for M-set: forward, 5′-GCGCGTTTTTGGTAGTTAATTC-3′ and reverse, 5′-CAAATTTTCCGCCATCGCTC-3′; for UM-set: forward, 5′-GTGTGTGTTTTTGGTAGTTAATTTG-3′ and reverse, 5′-CAAATTTTCCACCATCACTCAC-3′.

Bisulphite sequencing was performed with the primers common to methylated and unmethylated DNA sequences, using 1 µl sodium bisulphite-treated DNA. The PCR reaction was carried out using the following conditions: 1 cycle at 94°C for 5 min, 30 cycles at 94°C for 30 s, 57°C for 30 s, and 72°C for 1 min, and a final extension step at 72°C for 7 min. The PCR product was cloned into the TA vector pCR2.1 (Invitrogen). In total, 10 subclones were confirmed by restriction analysis and sequenced using the M13 forward primer (Invitrogen) with an ABI 3100 genetic analyser (Applied Biosystems). Primers sequences were forward, 5′-AATGGGGAGGAATGTTGTAGA-3′ and reverse, 5′-ATCTCCTAACTCCCCAACAAC-3′.

### Immunocytochemistry

For immunocytochemistry, cells were grown on eight-well chamber slides (1×10^5^ cells/well for LS174T and 2×10^4^ cells/well for all others) at 37°C in conventional culture medium, and fixed in 2% paraformaldehyde for 10 min at room temperature. Slides were incubated with 0.1% Triton X-100 buffered in phosphate-buffered saline (PBS) for 3 min, and washed three times with PBS at room temperature. Cells were blocked with 3% bovine serum albumin (BSA) in PBS for 1 h, and sequentially incubated with rabbit polyclonal anti-Mieap antibody (1∶200) or mouse monoclonal anti-LAMP1 antibody (1∶200) for 1 h at room temperature. After washing three times with PBS, slides were incubated with fluorescein isothiocyanate (FITC)-conjugated goat anti-rabbit immunoglobulin G (IgG) antibody, FITC-conjugated goat anti-mouse IgG antibody, Alexa Fluor 546 goat anti-rabbit IgG antibody, or Alexa Fluor 546 goat anti-mouse IgG antibody for 1 h at room temperature. Slides were treated with 1 µM TO-PRO-3 (Invitrogen) for 15 min to stain the nuclei, and then washed four times with PBS. Slides were mounted with VECTASHIELD H-1000 (Vector Laboratories), and observed under an Olympus IX70 (Olympus) inverted fluorescence microscope coupled with a Radiance 2000 laser-scanning confocal system (Bio-Rad).

### Establishment of p53-KD and Mieap-KD cell lines using RNA interference

We established p53-KD cell lines using LS174T, A549 and HepG2 cells, as described previously [27]. We also established Mieap-KD cell lines using LS174T, A549 and HepG2 cells. Mieap expression was inhibited in these cell lines by retroviral expression of short-hairpin RNA (shRNA) (Mieap-KD: 5′-gatccccGGAACTCACTCAAGCAGAAttcaagagaTTCTGCTTGAGTGAGTTCCtttttggaaa-3′) against the Mieap sequence. We also established control cell lines by infection with an empty retroviral vector (Control).

### DNA-damaging treatments

Cells were seeded 12 h before the treatment and were 60–70% confluent at the time of the treatment. To examine the expression of Mieap in response to genotoxic stresses, the cells were continuously treated with 1 mM or 100 µM H_2_O_2_ for 2 h, 1.0 µg/ml adriamycin for 2 h, or γ-irradiated at 60 Gy using a ^60^Co source. We confirmed that MALM was inducible by γ-irradiation at as low as 1 Gy (data not shown).

### Antibodies

Rabbits were immunized with the recombinant amino (N)-terminal domain of Mieap. Antibodies were subsequently purified on antigen affinity columns. The other primary antibodies used in this study were mouse monoclonal anti-beta-actin antibody (clone AC-74, Sigma), mouse monoclonal anti-p21/WAF1 antibody (Calbiochem), mouse monoclonal anti-ATP synthase alpha antibody (Invitrogen), rabbit polyclonal anti-ATP synthase beta antibody (Atlas antibody), mouse monoclonal anti-ATP synthase beta antibody (Novas biological), rabbit polyclonal anti-MTND1 antibody (Anova), mouse monoclonal anti-VDAC/Porine antibody (Abcam), mouse monoclonal anti-mitofilin antibody (Calbiochem), mouse monoclonal anti-cathepsin D antibody (Novus biological), goat polyclonal anti-cathepsin B antibody (R & D systems), mouse monoclonal anti-LAMP1 antibody (BD pharmingen), and mouse monoclonal anti-LAMP2 antibody (BD pharmingen).

### Visualization of subcellular organelles

Mitochondria, ER and Golgi were visualized by infection with recombinant adenovirus vectors or transfection with plasmids designed to express organelle marker proteins such as DsRed-Mito for mitochondria, DsRed-ER for ER and GFP-Golgi for Golgi. Alternatively, to visualize mitochondria, cells were incubated with 50 nM MitoTracker-Red (Invitrogen) or 50 nM MitoTracker-Green (Invitrogen) for 30 min at 37°C before fixation. To visualize lysosomes, 50 nM LysoTracker-Red (Invitrogen) for 30 min at 37°C before fixation.

### Transmission electron microscopy

The control and Mieap-KD cells of LS174T or A549 (1.5×10^5^ cells/24-well plate) or Ad-Mieap and Ad-LacZ infected HCT116 cells (6×10^4^ cells/24-well plate) were irradiated by γ ray at 60 Gy. On day 3 after IR, the cells were fixed in 2.5% glutaraldehyde in phosphate buffered saline (PBS) at 4°C for 2 h. The cells were washed with PBS, post-fixed in 1% OsO_4_ buffered with PBS for 2 h, dehydrated in a graded series of ethanol and embedded in Epon 812. Ultrathin sections (90 nm) were collected on copper grids, double-stained with uranyl acetate and lead citrate, and then observed using transmission electron microscopy (H-7100, Hitachi).

### Immunoelectron microscopy

For pre-embedding immunoelectron microscopic analysis with diaminobenzidine tetrahydrochloride (DAB), Ad-Mieap and Ad-LacZ infected HCT116 cells (6×10^4^ cells/24-well plate), or the control and Mieap-KD cells of LS174T or A549 (1.5×10^5^ cells/24-well plate) were irradiated by γ ray at 60 Gy. On day 3 after IR, the cells were fixed by 2% paraformaldehyde in PBS for 20 min and permeabilized with 0.1% Triton X-100 in PBS for 2 min at RT (room temperature), and then blocked with 3% bovine serum albumin. The cells were incubated with the primary antibodies including anti-Mieap, anti-LAMP1, anti-LAMP2, anti-cathepsin D, or anti-cathepsin B antibodies, for 12 h at 4°C, followed by incubation with Histofine Simple Stain MAX PO(M) or Histofine Simple Stain MAX PO(R) (Nichirei) for 2 h at RT. The immunoreactant was visualized with 0.05% DAB (Sigma) - 0.015% H_2_O_2_ in PBS for 5 min at RT. The cells were then postfixed with 1% OsO_4_ (Taab) in PBS for 2 h at 4°C. After postfixation the cells were dehydrated through a graded ethanol series and embedded in Epon 812 (Taab). Thin sections were cut with a diamond knife (Diatome) on an ultra-microtome (Leica) and examined with a transmission electron microscope H-7100 (Hitachi) at 75 kV.

For post-embedding immunoelectron microscopic analysis with gold particles, Ad-Mieap and Ad-LacZ infected HCT116 cells (1.5×10^6^ cells/10 cm dish) were irradiated by γ ray at 60 Gy. On day 3 after IR, the cells were fixed by 4% paraformaldehyde in PBS for 2 h at RT. The fixed cells were pelleted, and suspended in a quantity of 10% gelatin in PBS, which was equal to the volume of the pelleted cells. The gelatin-embedded cells were solidified on ice, and then incubated with PVP-sucrose cryoprotectant (20% polyvinylpyrrolidone, 1.84 M sucrose, 0.055 M Na_2_CO_3_, pH 7.4) for 24 h at 4°C. In order to obtain frozen ultrathin sections, the segmented blocks of gelatin-embedded cells were mounted on ultracyotome stubs, and frozen in liquid nitrogen. Ultrathin sections (90–100 nm) were cut using a Leica Ultracut S (Leica) with a cryoattachment, and lifted and fixed on a small drop of 2.3 M sucrose, and mounted on Formvar-coated nickel grids. In order to remove the sucrose on the sections, the grids were washed gently with 1.5% normal goat serum in PBS. Subsequently, the grids were washed four times with PBS, and incubated with 1.5% normal goat serum in PBS for 1 h at RT for blocking. After blocking, the sections were incubated with primary antibodies (1∶20) for 16 h at 4°C. The sections were washed six times with PBS, and incubated with goat anti-rabbit or goat anti-mouse 10 nm-colloidal gold-conjugated secondary antibodies (1∶20) for 2 h at RT. The sections were washed six times in PBS, and fixed by 2% glutaraldehyde in PBS for 5 min. The sections were washed four times in distilled H_2_O. The grids were embedded in a mixture containing 2.7% polyvinyl alcohol and 0.3% uranyl acetate. The sections on the grid were observed on a transmission electron microscope H-7100 (Hitachi) at 75 kV.

### Subcellular fractionation and proteinase K protection assay

LS174T-control and Mieap-KD cells or Ad-Mieap and Ad-LacZ infected HCT116 cells were irradiated by γ ray at 60 Gy. On day 3 after IR, to obtain crude mitochondrial fractions, these cells were suspended in cold homogenize buffer (20 mM HEPES pH 7.4, 250 mM sucrose) at 2×10^7^ cells/ml and homogenized by Dounce homogenization on ice. The homogenized samples were centrifuged at 800× g at 4°C for 10 min to pellet the nuclear fraction. The centrifugation was repeated, and the resulting supernatant was centrifuged at 10,000× g at 4°C for 10 min to obtain a mitochondrial pellet and cytosolic supernatant. The mitochondrial pellets were centrifuged again with fresh homogenization buffer and the pellets were suspended in Tris-HCl pH 7.4, 150 mM NaCl to react with protease, and subjected to proteinase K assay.

Lysosomes are usually co-fractionated in the mitochondrial pellet in this fractionation method. In order to eliminate the lysosomal proteins contaminated in the mitochondrial fraction, mitochondrial suspensions were pre-incubated with lysosomotropic detertgent (100 µM sphingosine, Sigma), 1% Triton X-100 or non-detergent at 37°C for 15 min. After incubation, these mitochondrial suspensions were treated with 0.2, 2, or 20 µg/ml proteinase K for 20 min on ice. Then, the proteinase K was inactivated with 2 mM PMSF for 10 min on ice. Cytosolic supernatants were also treated with 2 µg/ml proteinase K and inactivated by PMSF. These samples were dissolved into Laemmli buffer for SDS–PAGE and Western blots were performed with primary antibodies.

### Analysis of oxidized proteins

For detection of carbonyl-oxidized proteins, the Control and Mieap-KD cells of LS174T (3×10^6^ cells/6 cm-dish) were treated with 100 µM H_2_O_2_ for 2 h. Then, cell lysates were isolated at the indicated times. The supernatants were adjusted at 3 mg/ml protein extractions by Lowly-protein assay Kit (Bio-Rad). To derivatize the carbonyl groups on the protein side chains by 2,4-dinitrophenylhydrazine (DNPH), 5 µl of 12% SDS was added to 5 µl of protein extraction, and the mixture was incubated with 10 µl of 10 mM DNPH solution (OxyBlot kit, Chemicon), or 10 µl of control solution without DNPH for 15 min. After neutralization, the samples were subjected to the western blot analysis with rabbit polyclonal antibody against DNP-KLH (Invitrogen).

For detection of the carbonyl-oxidized form of the ATP synthase beta-subunit protein, the Control and Mieap-KD cells of LS174T (3×10^6^ cells/6 cm-dish) were treated with 100 µM H_2_O_2_ for 2 h (H_2_O_2_), irradiated by γ ray at 60 Gy (IR), or not treated (-). Then the cell lysates were isolated at day 5 after the treatment (H_2_O_2_ and IR) or immediately (non-treated (-)), and subjected to the immunoprecipitation (IP) experiment with mouse monoclonal anti-ATP synthase beta-subunit antibody (Novus) and control normal mouse IgG. The precipitates were washed five times in 1 ml lysis buffer (1% NP40, 150 mM NaCl, 25 mM Tris-HCl pH 7.6, complete protease inhibitor cocktail (Roche)), and then the precipitated ATP synthase beta subunit was released from the immunocomplexes by incubation with 30 µl of 12% SDS for 30 min at RT, and then, the supernatant was collected by centrifugation. To derivatize the carbonyl groups on the precipitated ATP synthase beta-subunit with DNPH, 10 µl supernatant solutions were incubated with 10 µl of 10 mM DNPH solution (OxyBlot kit, Chemicon) or 10 µl of control solution without DNPH for 15 min. After neutralization, the samples were subjected to the western blot analysis with rabbit polyclonal antibody against DNP-KLH (Invitrogen). For detection of total ATP synthase beta-subunit protein, the same membrane was stripped and re-immunoprobed by rabbit polyclonal antibody against ATP synthase beta-subunit (Atlas).

For detection of 3-nitrotyrosine oxidized proteins, the Control and Mieap-KD cells of LS174T and the Ad-Mieap and Ad-LacZ infected cells of HCT116 were treated with 1 mM H_2_O_2_ for 2 h (H_2_O_2_), irradiated by γ ray at 60 Gy (IR), or not treated (-). Then, day 1 after the H_2_O_2_ treatment, day 3 after IR, or immediately (non-treatment (-)), the cells were subjected to IF experiment with rabbit polyclonal anti-nitrotyrosine antibody. The cells on 8-well chamber slides were fixed and blocked, and incubated with rabbit polyclonal antibody against nitrotyrosine (1∶100, Upstate) in blocking solution for 1.5 h. After washing with PBS, the cells were incubated with FITC conjugated goat anti-rabbit IgG secondary antibody (1∶300, Santa cruz) for 1 h. Nucleus and mitochondria in the cells were counterstained with TO-PRO-3 and DsRed-Mito, respectively. The nitrotyrosine signals in 300–500 cells were analyzed by confocal microscopy. The intensity of nitrotyrosine in each captured image was analyzed by LuminaVision image analysis software (Version 2.4). The total areas of nitrotyrosine in 300–400 cells were extracted using the optimal threshold parameters and calculated with LuminaVision image analysis software. The nitrotyrosine intensity was presented as the average of the calculated values per cell with error bars.

### Assay of mitochondrial ATP synthesis

The experiment was carried out according to the procedure reported previously [23]. The Control and Mieap-KD cells of LS174T (3×10^6^ cells/6 cm dish) or Ad-Mieap and Ad-LacZ infected cells of HCT116 (1×10^6^ cells/6 cm dish) were irradiated by γ ray at 30 Gy, and harvested day 7 after IR. The cells with or without the treatment were subjected to ATP synthesis assay as following. The cells (5×10^5^ cells) were collected by centrifugation at 900× g at room temperature (RT), and the cell pellet was resuspended in 160 µl of buffer A (150 mM KCl, 25 mM Tris-HCl, 2 mM EDTA, 10 mM KH_2_PO_4_, 0.1 mM MgCl_2_, 0.1% BSA, pH 7.4). 5 µl of digitonin (825 µg/ml) (Sigma) was added to the suspension (to 25 µg/ml), and incubated for 1 min at RT with gentle agitation. Digitonin was removed by washing the cells with 1 ml buffer A, and then centrifuged at 900× g at RT. The cell pellet was resuspended in 175 µl of buffer A. 5 ml of 6 mM *P^1^*, *P^5^* - di (adenosine) pentaphosphate (to 0.15 mM), 5 ml of 4 mM ADP (to 0.1 mM), 2.5 ml of 80 mM malate (to 1 mM) and 2.5 µl of 80 mM pyruvate (to 1 mM), and then 10 ml of buffer B (0.8 mM luciferin (Wako) and 20 µg/ml luciferase (Wako) in 0.5 M Tris-acetate, pH 7.75) were added to the cell suspension. Cell suspension was transferred to a luminometer cuvette. After a gentle mixing with a vortex for 2 s, the cuvette was placed in luminometer Rumat LB 9507 (Berthold technologies) and light emission was continuously recorded for 5 min (17 readings). To obtain the baseline luminescence activity corresponding to non-mitochondrial ATP production, light emission was recorded in the presence of oligomycin (to 4 µg/ml). The change in RLU (Relative light units) was then converted to ATP concentration based on an ATP standard curve. The activity was shown as ATP production per min in a cell (nmol/min/cell). The value was divided by the mean intensity of mitochondria (MitoTracker-Green) per cell in order to reflect the ATP synthesis activity of the same amount of mitochondria in a cell (nmol/min/mito/cell).

### Quantitative analysis of ROS

ROS generation by mitochondria in living cells was analyzed with the mitochondrial superoxide indicator MitoSOX-Red (Invitrogen) or dihydrorhodamine123 (DHR123) (Sigma). The cells were seeded onto a 35-mm glass-bottomed dish (Biousing Biotechnology) (1×10^5^ cells/well for LS174T and 2×10^4^ cells/well for all others) at 37°C in conventional media. After 24 h, the cells were treated with or without stresses, and the ROS level was examined at the indicated times. Briefly, the cells were incubated with 5 µM MitoSOX-Red or 10 µM DHR123 for 10 min at 37°C in serum-free media, washed twice with serum-free media, and then incubated with 5 µM CellMask (Invitrogen) for 15 min to visualize the walls of living cells. After washing twice with serum free-media, the stained cells were immediately observed under the confocal laser-scanning microscope, and the images were captured using an excitation filter of 510 nm and an emission filter of 580 nm for MitoSOX-Red, and an excitation filter of 543 nm and an emission filter of 579 nm for DHR123. The intensity of MitoSox-Red or DHR123 in each captured image was analyzed by LuminaVision image analysis software (Version 2.4). The total areas of MitoSOX-Red or DHR123 intensity in 300–400 cells were extracted using the optimal threshold parameters and calculated with LuminaVision image analysis software. The MitoSOX-Red or DHR123 intensity was presented as the average of the calculated values per cell with error bars.

## Supporting Information

Figure S1 **The Mieap promoter is methylated in Mieap-silenced cancer.** (A) Bisulfite sequencing confirmed that the promoter of Mieap is methylated in U373MG cells, but not in HepG2 cells as indicated by the MSP experiment. (B) HCT116 cell line was treated with the demethylating agent 5-aza-2′-deoxycytidine (5-Aza-dC) at a concentration of 1 µmol/L and 5 µmol/L (Sigma-Aldrich, St. Louis, MO). The cells were harvested 1 week after the treatment, and then, the expression level of *Mieap* mRNA was examined in the cells by RT- PCR and real-time PCR.
(TIF)

Figure S2 **Frequent methylation of the** ***Mieap*** **promoter and resulting repression of** ***Mieap*** **in human cancers.** The status upon methylation and expression of *Mieap* was examined in 10 human cancer cell lines by MSP and RT-PCR, respectively. Expression was examined in each cell infected (+) or not infected (−) with Ad-p53. In >60% (7/10) of the cancers, the promoter of *Mieap* was methylated, leading to repression of *Mieap*. Expression levels of autophagy-related genes, such as *ATG5*, *ATG12*, *BECN1* and *DRAM*, are shown as controls. U: unmethylated, M: methylated.
(TIF)

Figure S3 **Mieap induces colocalization of lysosomes with mitochondria.** (A) Exogenous Mieap induces overlapping of lysosomes and mitochondria. Three cancer cell lines (HepG2, H1299 and U373MG) were infected with Ad-Mieap or Ad-LacZ at an MOI of 5, and subjected to immunofluorescence (IF) analysis 24 h after infection. Mieap protein was stained with polyclonal rabbit anti-Mieap antibody (Mieap: green or red). Lysosomes were stained with anti-LAMP1 antibody (LAMP1: green). Mitochondria were indicated by the DsRed-mito protein signal (Mito: red). Scale bars = 20 µm (B) The phenomenon is deficient in Mieap-methylated cancers. The Mieap-methylated cancers, H1299 and U373MG, were irradiated by γ ray, and at the indicated times, the cells were subjected to IF analysis. Lysosomes were stained with anti-LAMP1 antibody (LAMP1: green). Mitochondria were indicated by the DsRed-mito protein signal (Mito: red). Scale bars = 20 µm.
(TIF)

Figure S4 **Lysotracker specifically targets Mieap-induced mitochondrial acidic compartments.** HCT116 cells (A) and A549 cells (B) were seeded on 8-well chamber slides (2×10^4^ cells/well) at 37°C in conventional media. After 24 h, HCT116 cells or A549 cells were infected with both Ad-Mieap at an MOI of 5 and Ad-GFP-Mito at an MOI of 30 or only Ad-GFP-Mito at an MOI of 30, respectively. 24 h after infection, the cells were irradiated by γ ray, and 3 days after irradiation, the cells were or were not treated with 25 mM NH4Cl for 2 h. After the treatment, the cells were subjected to immunocytochemical experiment with Lysotracker.
(TIF)

Figure S5 **Anti-LAMP1 and LAMP2 antibodies specifically detect lysosomes indicated by LysoTracker-Red or anti-cathepsin D antibody.** HCT116 were seeded on 8-well chamber slides (2×10^4^ cells/well) at 37°C in conventional media. 24 h before IR, HCT116 cells were infected with Ad-LacZ at an MOI of 5, and the cells were irradiated by γ ray, and 3 days after IR, the cells were subjected to immunocytochemical experiment with anti-LAMP1, anti-LAMP2, anti-cathepsin D antibodies, and LysoTracker-Red.
(TIF)

Figure S6 **Endogenous Mieap induces colocalization of lysosomes with mitochondria in LS174T cells.** The cont and Mieap-KD cells of LS174T were subjected to IF experiment on day 3 after IR. Mieap protein was stained with polyclonal rabbit anti-Mieap antibody (Mieap: green or red). Lysosomes were stained with anti-LAMP1 antibody (LAMP1: green). Mitochondria were indicated by the DsRed-mito protein signal (Mito: red). Scale bars = 10 µm.
(TIF)

Figure S7 **Electron Microscopic analysis of the cont and Mieap-KD cells of A549.** The cont and Mieap-KD cells of A549 were subjected to electron microscopic analysis on day 3 after IR. The representative images are shown. N: nucleus m: mitochondria A: actin Scale bar = 10 µm (upper), 1 µm (middle), or 500 nm (lower).
(TIF)

Figure S8 **Electron Microscopic analysis of the cont and Mieap-KD cells of LS174T.** The cont and Mieap-KD cells of LS174T were subjected to electron microscopic analysis on day 3 after IR. The representative images are shown. N: nucleus m: mitochondria Scale bar = 2 µm (upper) or 500 nm (middle and lower).
(TIF)

Figure S9 **LC3-positive autophagosomes are not related to the Mieap-regulated lysosomes.** The Ad-LacZ and Ad-Mieap infected cells of HCT116, the cont and Mieap-KD cells of A549, or the cont and Mieap-KD cells of LS174T were subjected to IF analysis on day 3 after IR. Autophagosomes were indicated by the dot signal of GFP-LC3 (LC3: green). Mieap protein was stained with polyclonal rabbit anti-Mieap antibody (Mieap: red). Lysosomes were stained with anti-LAMP1 antibody (LAMP1: red). Mitochondria were indicated by the DsRed-mito protein signal (Mito: red). Scale bars = 20 µm.
(TIF)

Figure S10 **Pre-embedding immunoelectron microscopic analysis on Mieap.** The Ad-LacZ and Ad-Mieap infected cells of HCT116 were subjected to pre-embedding immunoelectron microscopic analysis using DAB and anti-Mieap antibody on day 3 after IR. The representative images are shown. Arrowheads (yellow) indicate representative mitochondria. N: nucleus Scale bar = 10 µm (upper) or 500 nm (lower).
(TIF)

Figure S11 **Pre-embedding immunoelectron microscopic analysis on cathepsin D.** The Ad-LacZ and Ad-Mieap infected cells of HCT116 were subjected to pre-embedding immunoelectron microscopic analysis using DAB and anti-cathepsin D antibody on day 3 after IR. The representative images are shown. Arrowheads (yellow) indicate representative mitochondria. N: nucleus Scale bar = 10 µm (upper) or 500 nm (lower).
(TIF)

Figure S12 **Pre-embedding immunoelectron microscopic analysis on LAMP1.** The Ad-LacZ and Ad-Mieap infected cells of HCT116 were subjected to pre-embedding immunoelectron microscopic analysis using DAB and anti-LAMP1 antibody on day 3 after IR. The representative images are shown. Arrowheads (yellow) indicate representative mitochondria. N: nucleus CL: cytoplasmic lysosome Scale bar = 10 µm (upper) or 500 nm (lower).
(TIF)

Figure S13 **Pre-embedding immunoelectron microscopic analysis on LAMP2.** The Ad-LacZ and Ad-Mieap infected cells of HCT116 were subjected to pre-embedding immunoelectron microscopic analysis using DAB and anti-LAMP2 antibody on day 3 after IR. The representative images are shown. Arrowheads (yellow) indicate representative mitochondria. N: nucleus Scale bar = 10 µm (upper), 2 µm (middle), or 1 µm (lower).
(TIF)

Figure S14 **Pre-embedding immunoelectron microscopic analysis on cathepsin B.** The Ad-LacZ and Ad-Mieap infected cells of HCT116 were subjected to pre-embedding immunoelectron microscopic analysis using DAB and anti-cathepsin B antibody on day 3 after IR. The representative images are shown. Arrowheads (yellow) indicate representative mitochondria. N: nucleus CL: cytoplasmic lysosome Scale bar = 10 µm (upper), 2 µm (middle), or 500 nm (lower).
(TIF)

Figure S15 **Post-embedding immunoelectron microscopic analysis on Mieap.** The Ad-LacZ and Ad-Mieap infected cells of HCT116 were subjected to post-embedding immunoelectron microscopic analysis using gold particles and anti-Mieap antibody on day 3 after IR. The representative images are shown. The broken black line indicates the mitochondrial region. The red line indicates the mitochondrial cristae region. Scale bar = 100 nm or 200 nm.
(TIF)

Figure S16 **Post-embedding immunoelectron microscopic analysis on cathepsin D and LAMP1.** The Ad-LacZ and Ad-Mieap infected cells of HCT116 were subjected to post-embedding immunoelectron microscopic analysis using gold particles, anti-cathepsin D antibody, and anti-LAMP1 antibody on day 3 after IR. The representative images are shown. The broken black line indicates the mitochondrial region. The red line indicates the mitochondrial cristae region. Scale bar = 100 nm or 200 nm.
(TIF)

Figure S17 **Whole image of western blot analysis on intramitochondrial and cytoplasmic proteins of cathepsin D and cathepsin B.** The whole image of western blot analysis on intramitochondrial and cytoplasmic proteins of cathepsin D (A) and cathepsin B (B) in Figure 7 is shown.
(TIF)
