# Supplementary figures and images for: Possible Existence of Lysosome-Like Organella within Mitochondria and Its Role in Mitochondrial Quality Control
Source: PLoS One. 2011 Jan 17;6(1):e16054. doi: 10.1371/journal.pone.0016054 (PMC3022026; doi:10.1371/journal.pone.0016054)

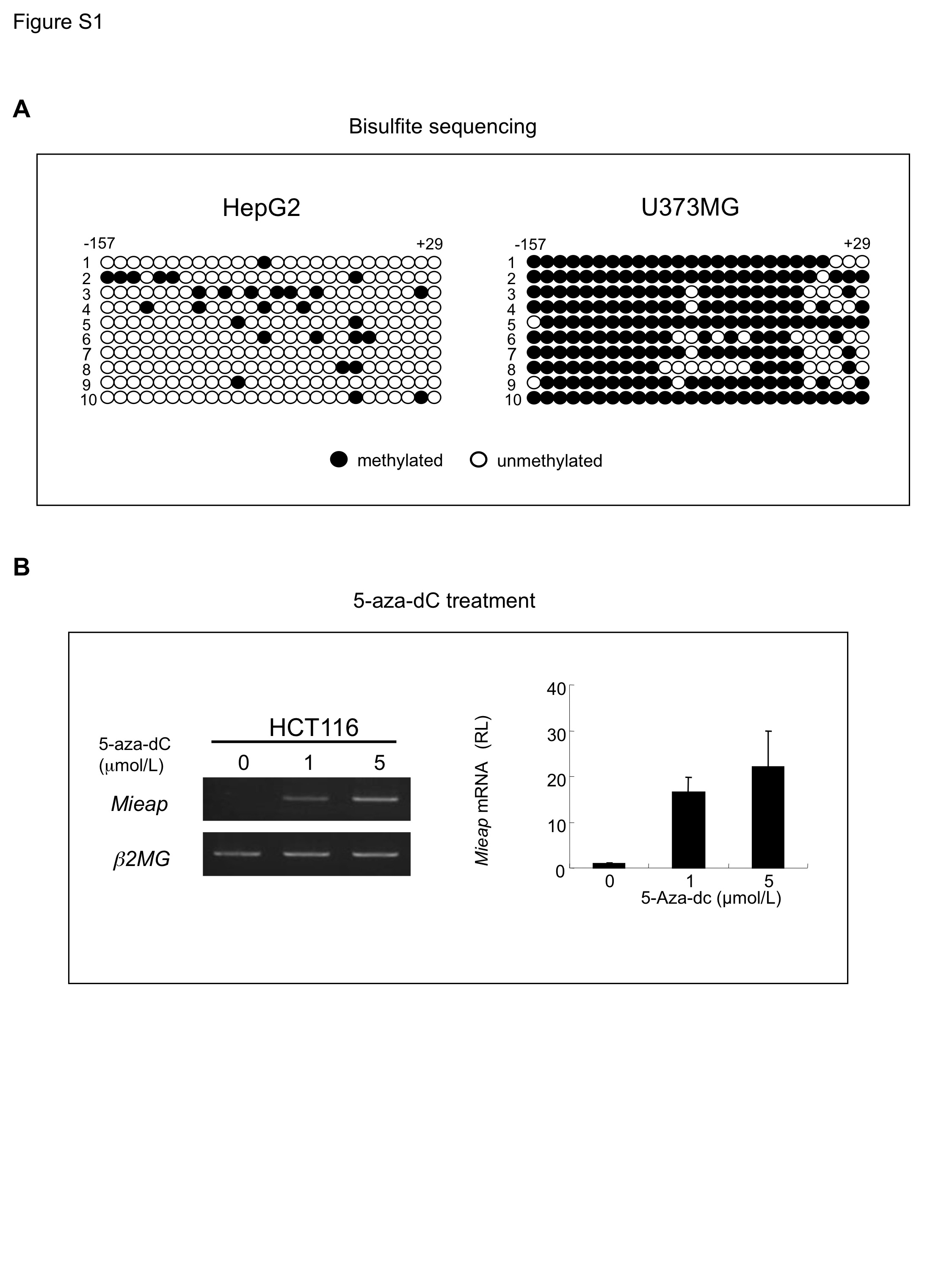

Supplement: Figure S1 — The Mieap promoter is methylated in Mieap-silenced cancer. (A) Bisulfite sequencing confirmed that the promoter of Mieap is methylated in U373MG cells, but not in HepG2 cells as indicated by the MSP experiment. (B) HCT116 cell line was treated with the demethylating agent 5-aza-2′-deoxycytidine (5-Aza-dC) at a concentration of 1 µmol/L and 5 µmol/L (Sigma-Aldrich, St. Louis, MO). The cells were harvested 1 week after the treatment, and then, the expression level of Mieap mRNA was examined in the cells by RT- PCR and real-time PCR. (TIF) [file pone.0016054.s001.tif]

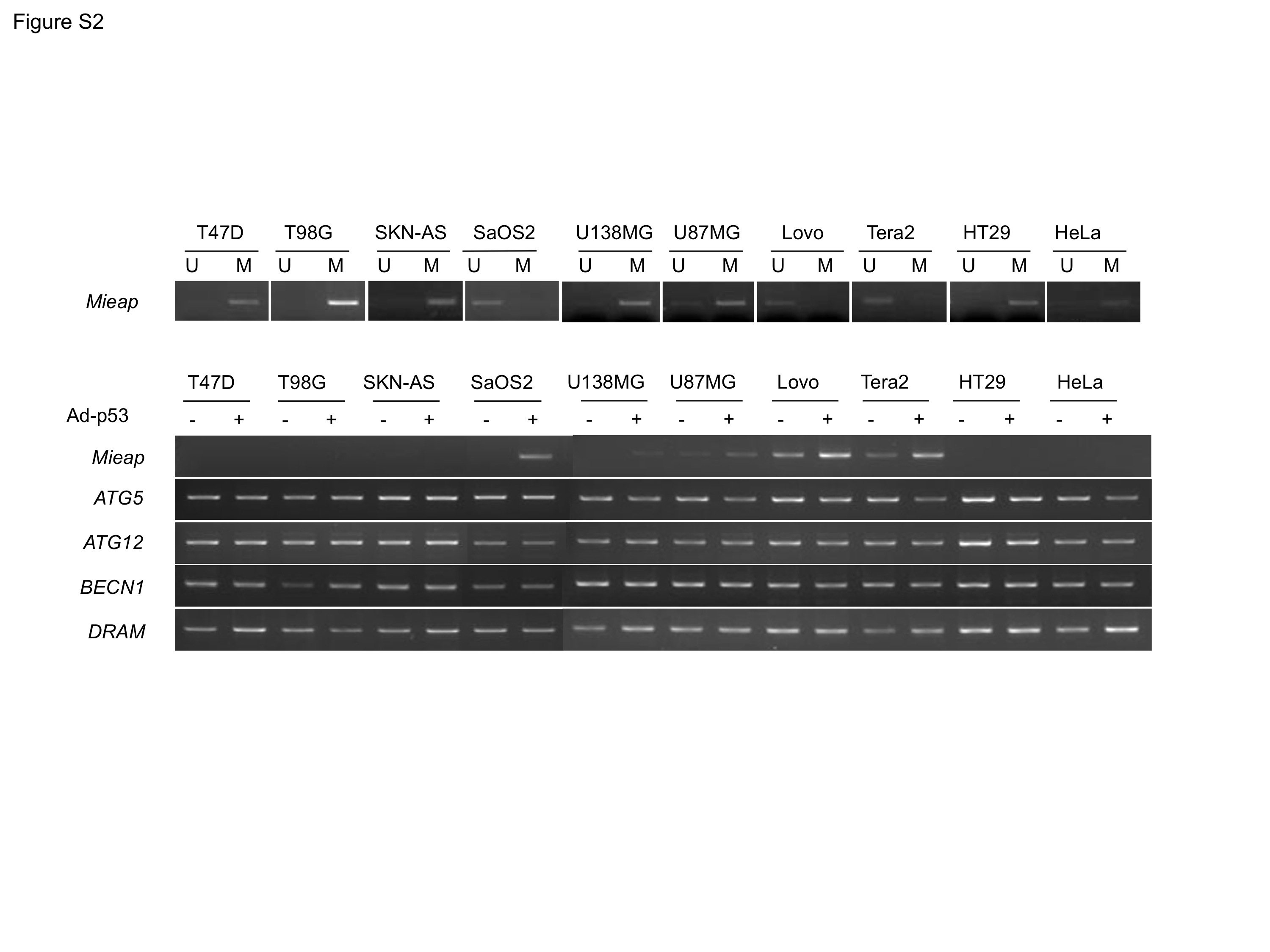

Supplement: Figure S2 — Frequent methylation of the Mieap promoter and resulting repression of Mieap in human cancers. The status upon methylation and expression of Mieap was examined in 10 human cancer cell lines by MSP and RT-PCR, respectively. Expression was examined in each cell infected (+) or not infected (−) with Ad-p53. In >60% (7/10) of the cancers, the promoter of Mieap was methylated, leading to repression of Mieap. Expression levels of autophagy-related genes, such as ATG5, ATG12, BECN1 and DRAM, are shown as controls. U: unmethylated, M: methylated. (TIF) [file pone.0016054.s002.tif]

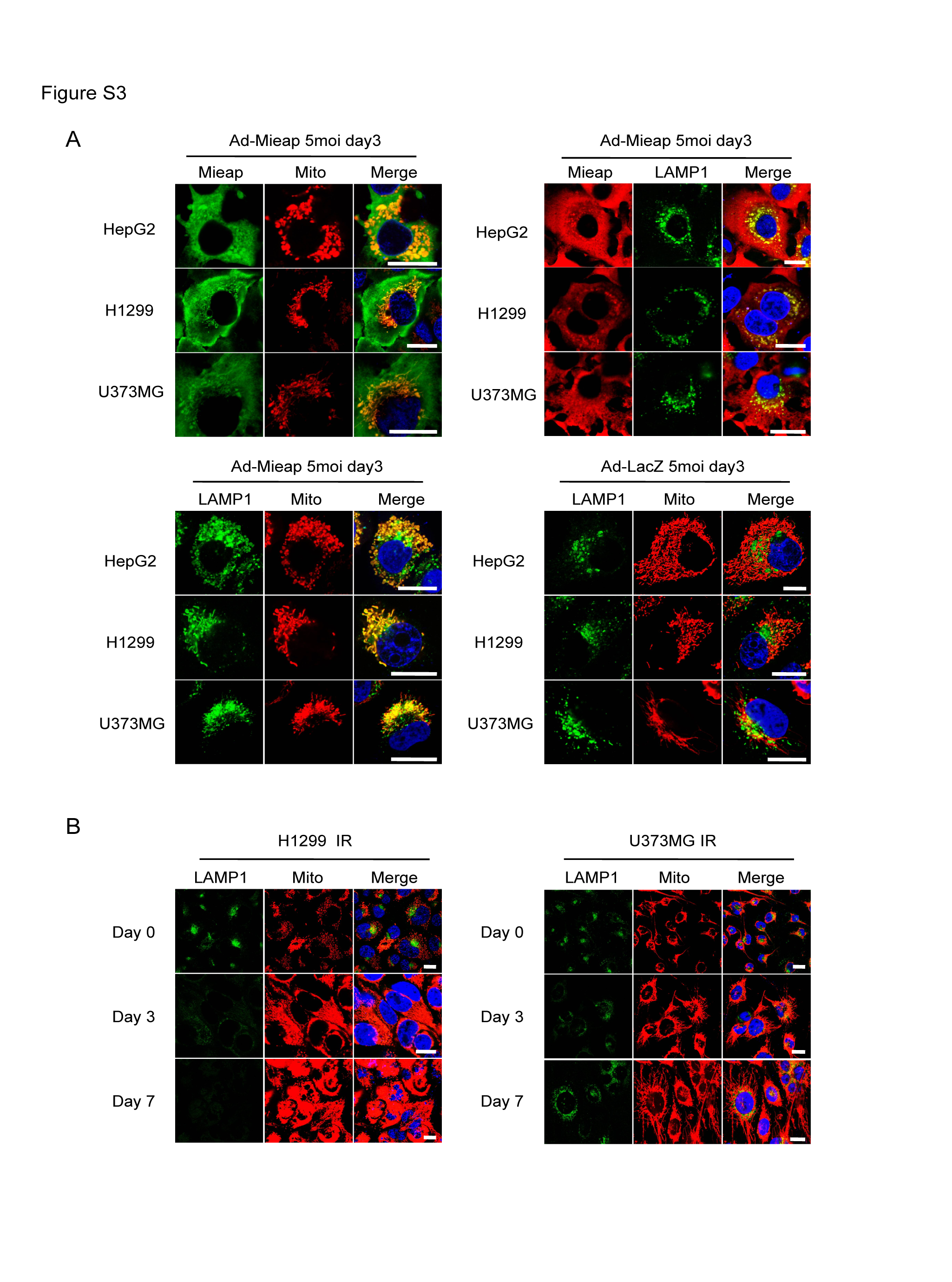

Supplement: Figure S3 — Mieap induces colocalization of lysosomes with mitochondria. (A) Exogenous Mieap induces overlapping of lysosomes and mitochondria. Three cancer cell lines (HepG2, H1299 and U373MG) were infected with Ad-Mieap or Ad-LacZ at an MOI of 5, and subjected to immunofluorescence (IF) analysis 24 h after infection. Mieap protein was stained with polyclonal rabbit anti-Mieap antibody (Mieap: green or red). Lysosomes were stained with anti-LAMP1 antibody (LAMP1: green). Mitochondria were indicated by the DsRed-mito protein signal (Mito: red). Scale bars = 20 µm (B) The phenomenon is deficient in Mieap-methylated cancers. The Mieap-methylated cancers, H1299 and U373MG, were irradiated by γ ray, and at the indicated times, the cells were subjected to IF analysis. Lysosomes were stained with anti-LAMP1 antibody (LAMP1: green). Mitochondria were indicated by the DsRed-mito protein signal (Mito: red). Scale bars = 20 µm. (TIF) [file pone.0016054.s003.tif]

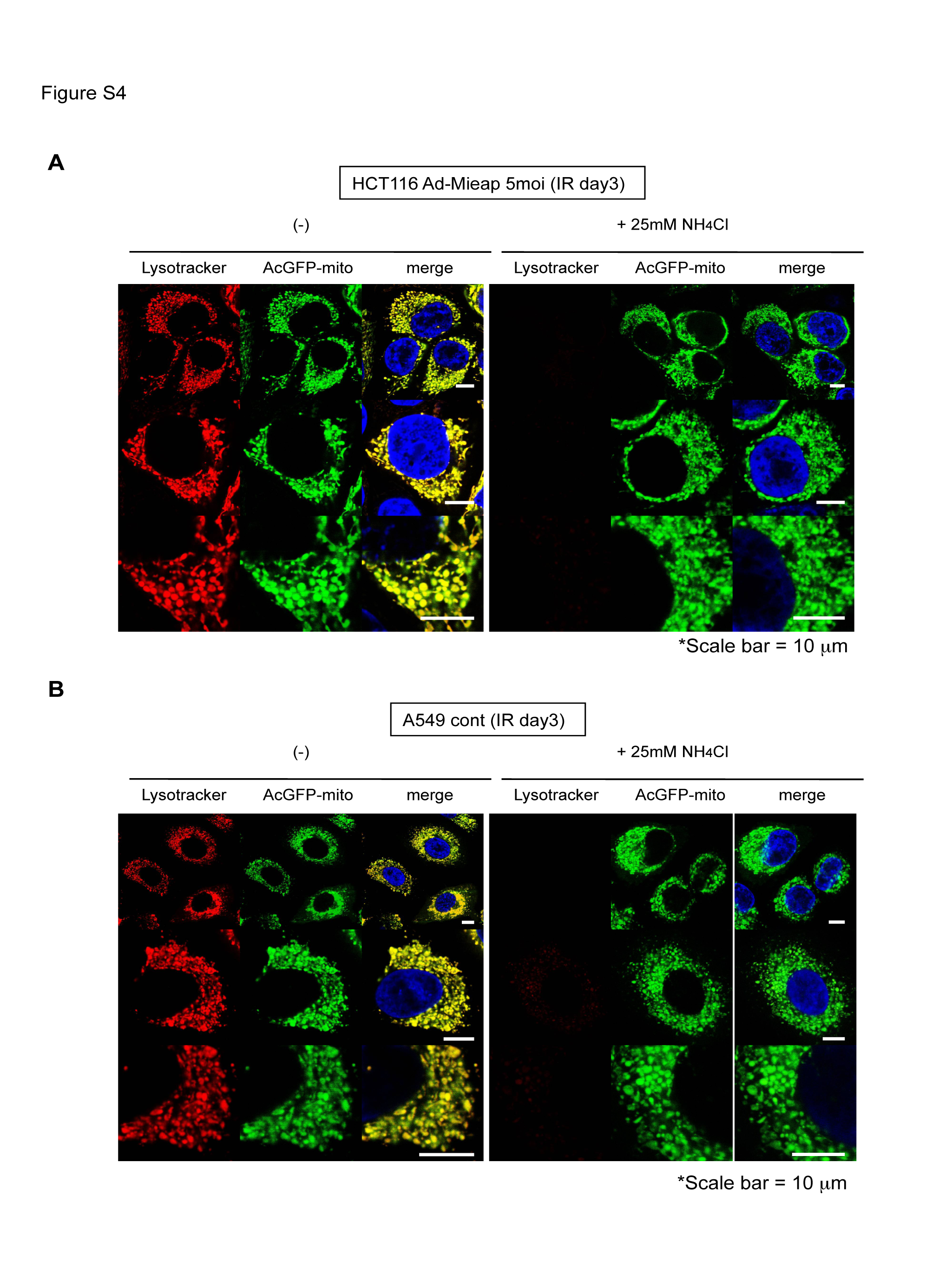

Supplement: Figure S4 — Lysotracker specifically targets Mieap-induced mitochondrial acidic compartments. HCT116 cells (A) and A549 cells (B) were seeded on 8-well chamber slides (2×10^4^ cells/well) at 37°C in conventional media. After 24 h, HCT116 cells or A549 cells were infected with both Ad-Mieap at an MOI of 5 and Ad-GFP-Mito at an MOI of 30 or only Ad-GFP-Mito at an MOI of 30, respectively. 24 h after infection, the cells were irradiated by γ ray, and 3 days after irradiation, the cells were or were not treated with 25 mM NH4Cl for 2 h. After the treatment, the cells were subjected to immunocytochemical experiment with Lysotracker. (TIF) [file pone.0016054.s004.tif]

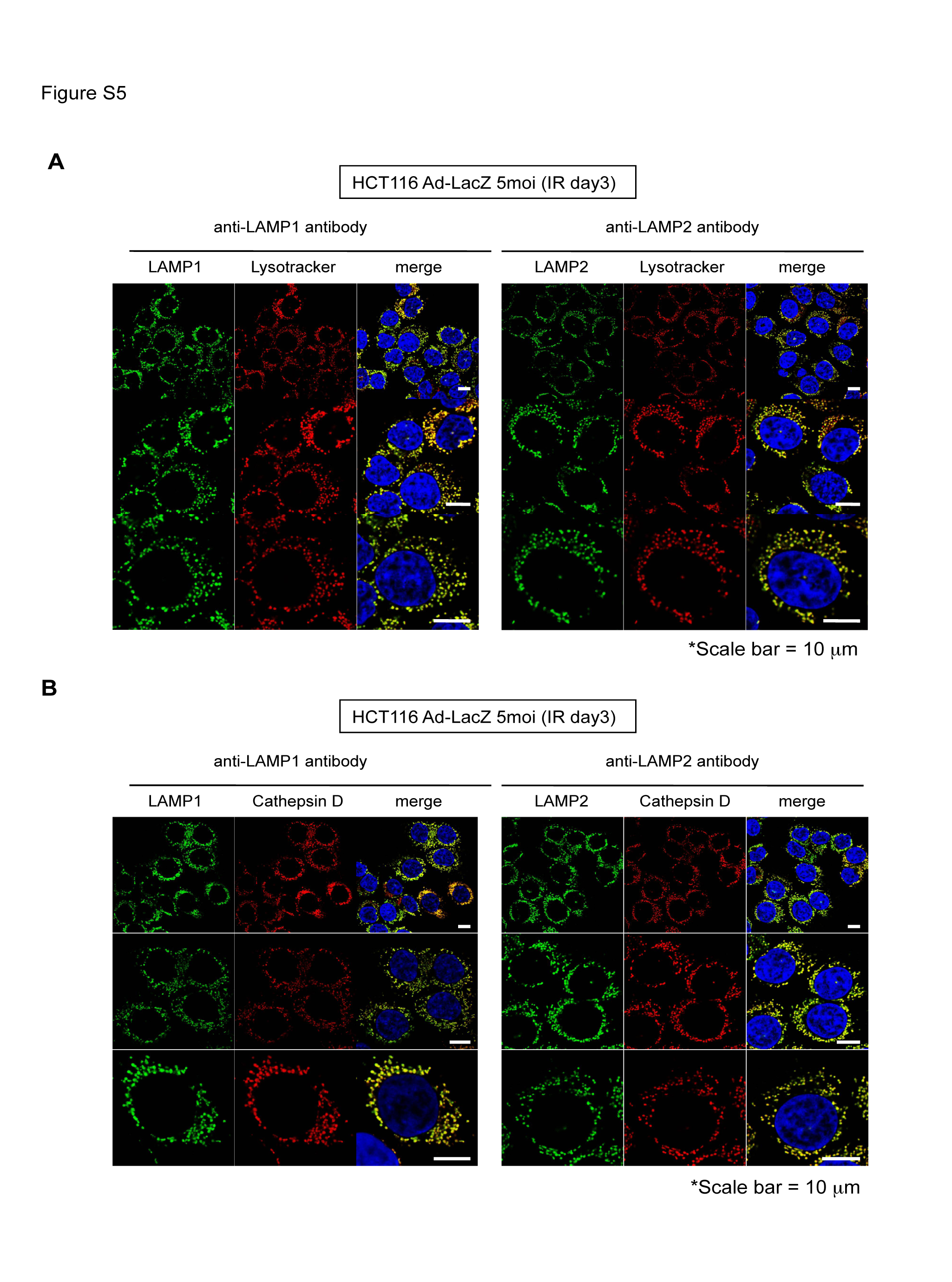

Supplement: Figure S5 — Anti-LAMP1 and LAMP2 antibodies specifically detect lysosomes indicated by LysoTracker-Red or anti-cathepsin D antibody. HCT116 were seeded on 8-well chamber slides (2×10^4^ cells/well) at 37°C in conventional media. 24 h before IR, HCT116 cells were infected with Ad-LacZ at an MOI of 5, and the cells were irradiated by γ ray, and 3 days after IR, the cells were subjected to immunocytochemical experiment with anti-LAMP1, anti-LAMP2, anti-cathepsin D antibodies, and LysoTracker-Red. (TIF) [file pone.0016054.s005.tif]

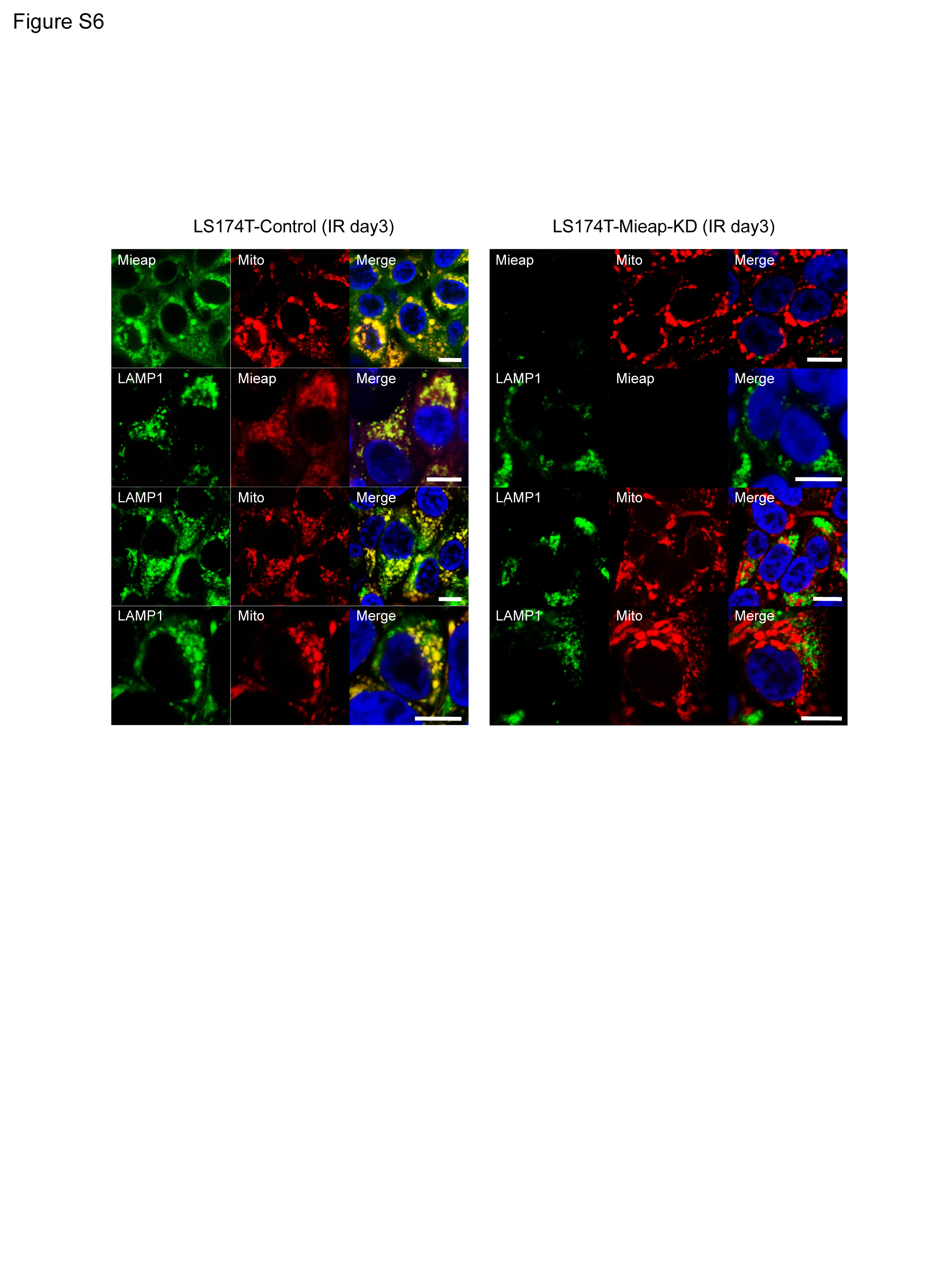

Supplement: Figure S6 — Endogenous Mieap induces colocalization of lysosomes with mitochondria in LS174T cells. The cont and Mieap-KD cells of LS174T were subjected to IF experiment on day 3 after IR. Mieap protein was stained with polyclonal rabbit anti-Mieap antibody (Mieap: green or red). Lysosomes were stained with anti-LAMP1 antibody (LAMP1: green). Mitochondria were indicated by the DsRed-mito protein signal (Mito: red). Scale bars = 10 µm. (TIF) [file pone.0016054.s006.tif]

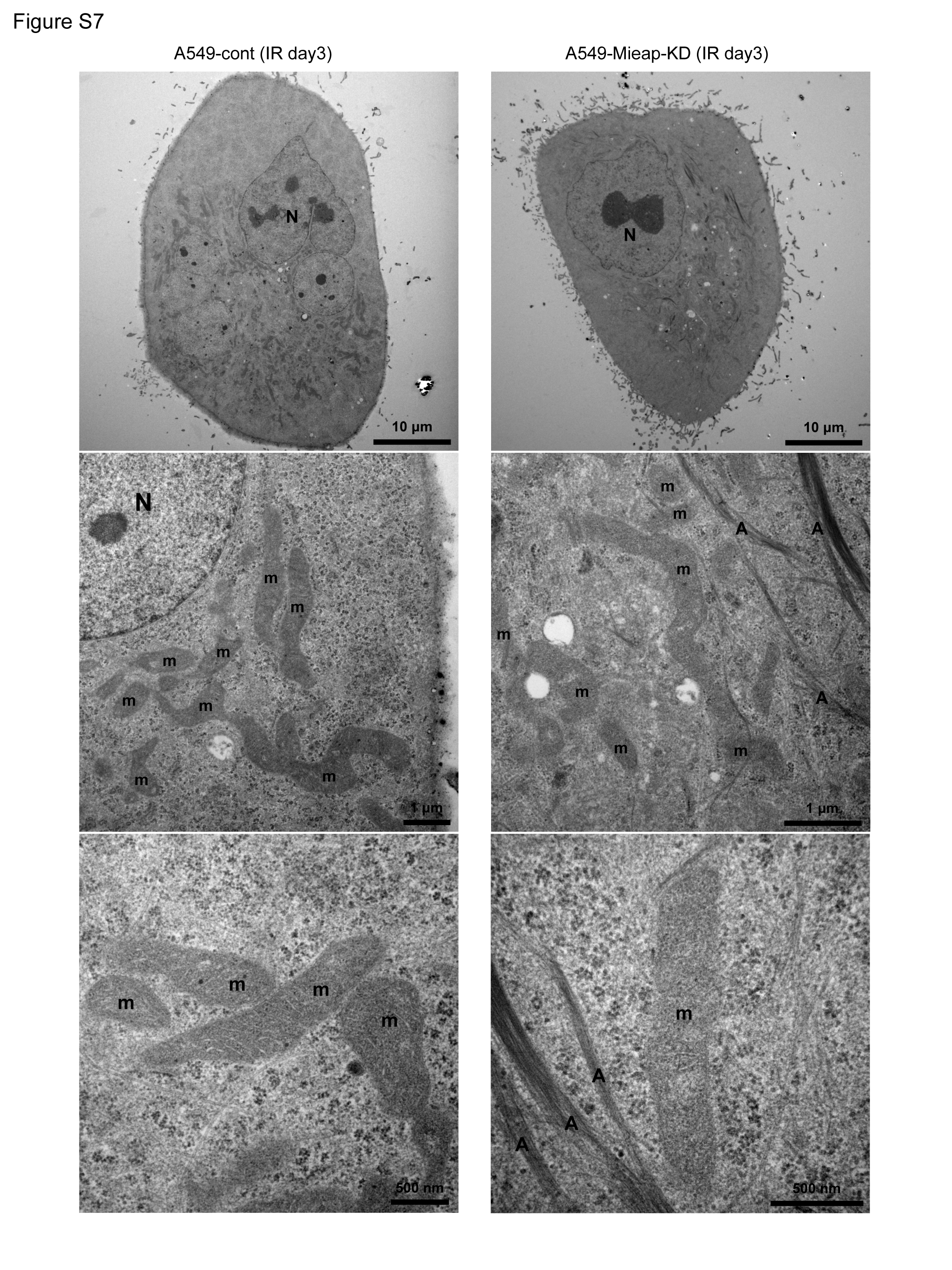

Supplement: Figure S7 — Electron Microscopic analysis of the cont and Mieap-KD cells of A549. The cont and Mieap-KD cells of A549 were subjected to electron microscopic analysis on day 3 after IR. The representative images are shown. N: nucleus m: mitochondria A: actin Scale bar = 10 µm (upper), 1 µm (middle), or 500 nm (lower). (TIF) [file pone.0016054.s007.tif]

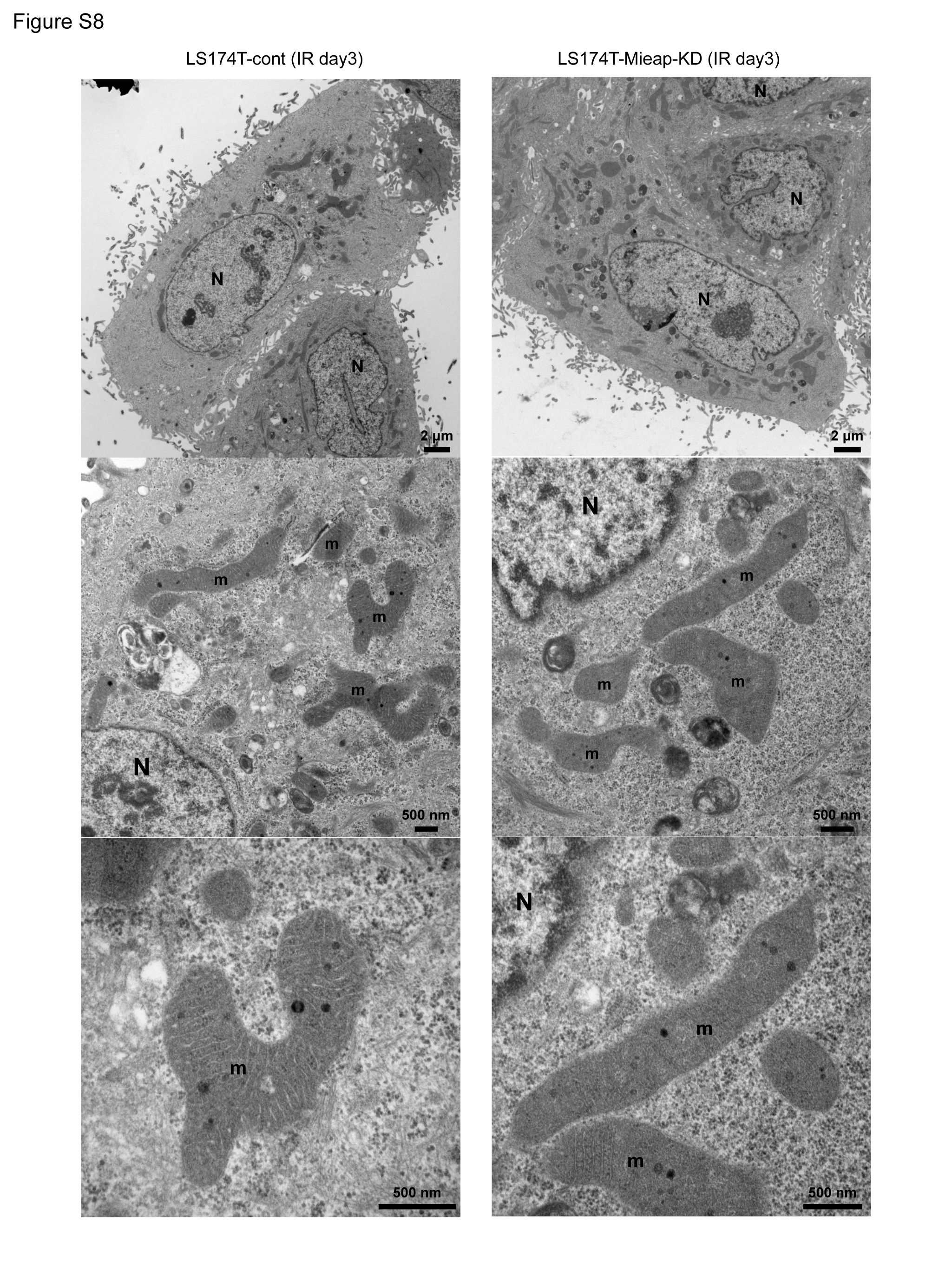

Supplement: Figure S8 — Electron Microscopic analysis of the cont and Mieap-KD cells of LS174T. The cont and Mieap-KD cells of LS174T were subjected to electron microscopic analysis on day 3 after IR. The representative images are shown. N: nucleus m: mitochondria Scale bar = 2 µm (upper) or 500 nm (middle and lower). (TIF) [file pone.0016054.s008.tif]

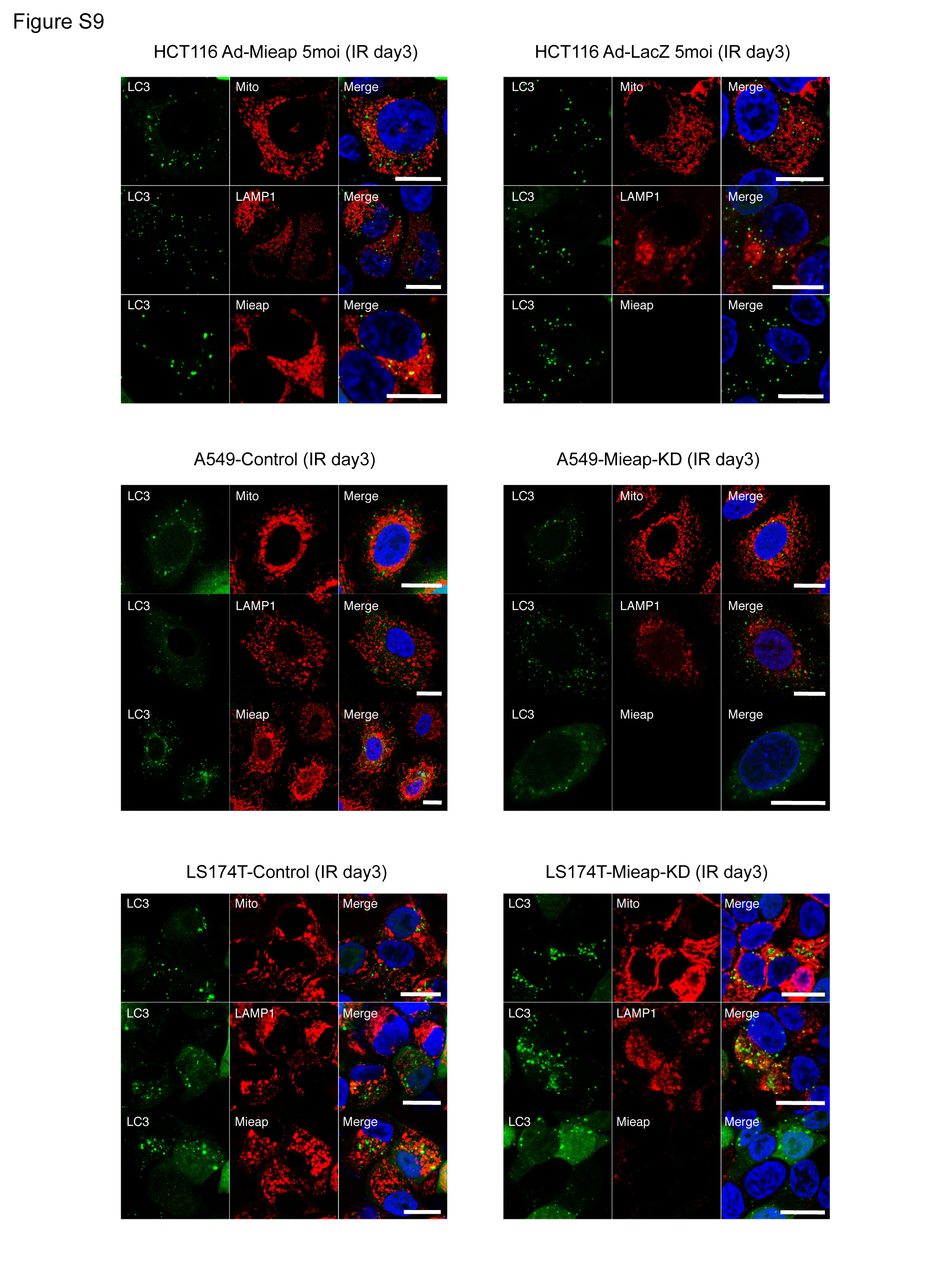

Supplement: Figure S9 — LC3-positive autophagosomes are not related to the Mieap-regulated lysosomes. The Ad-LacZ and Ad-Mieap infected cells of HCT116, the cont and Mieap-KD cells of A549, or the cont and Mieap-KD cells of LS174T were subjected to IF analysis on day 3 after IR. Autophagosomes were indicated by the dot signal of GFP-LC3 (LC3: green). Mieap protein was stained with polyclonal rabbit anti-Mieap antibody (Mieap: red). Lysosomes were stained with anti-LAMP1 antibody (LAMP1: red). Mitochondria were indicated by the DsRed-mito protein signal (Mito: red). Scale bars = 20 µm. (TIF) [file pone.0016054.s009.tif]

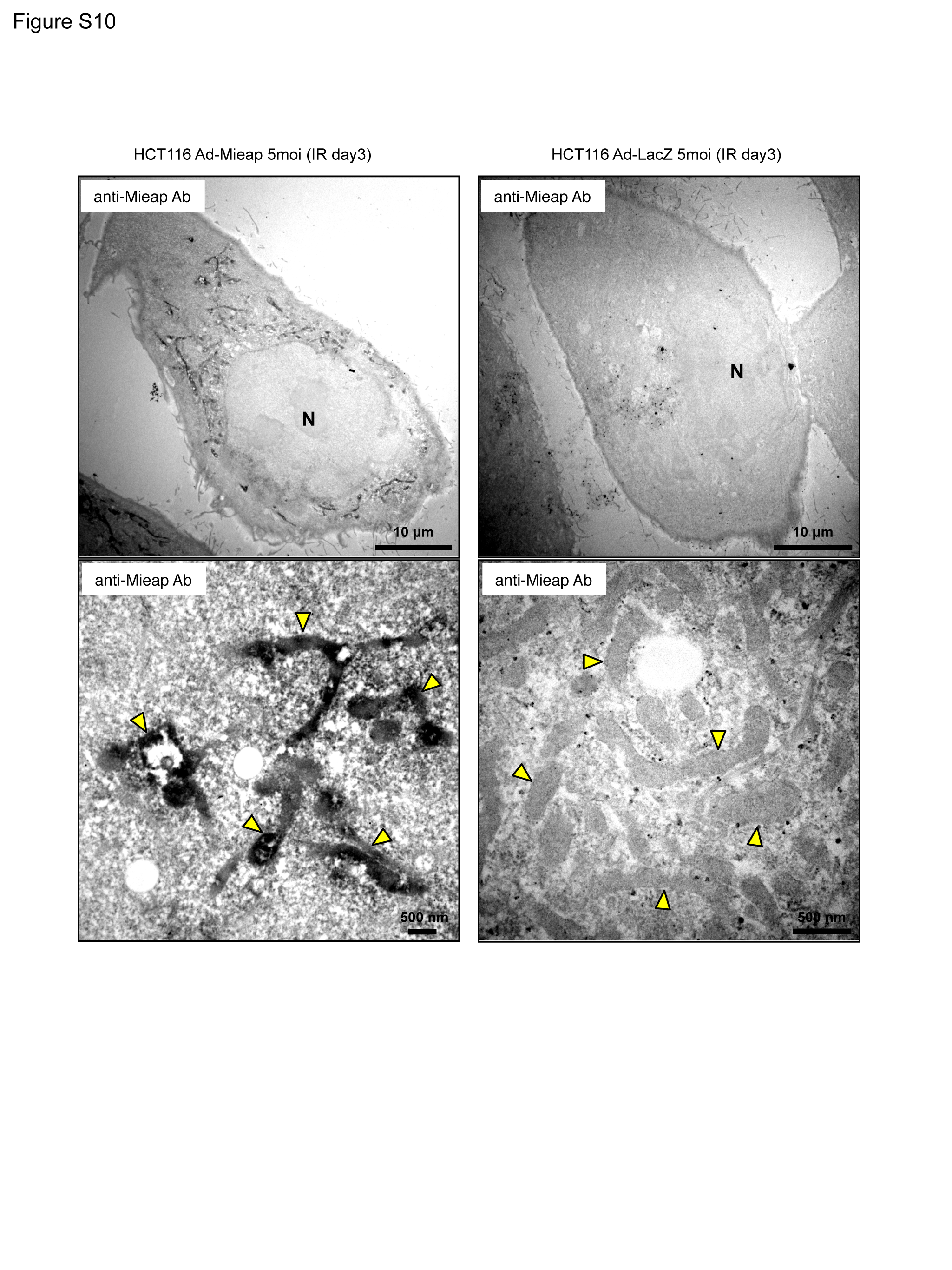

Supplement: Figure S10 — Pre-embedding immunoelectron microscopic analysis on Mieap. The Ad-LacZ and Ad-Mieap infected cells of HCT116 were subjected to pre-embedding immunoelectron microscopic analysis using DAB and anti-Mieap antibody on day 3 after IR. The representative images are shown. Arrowheads (yellow) indicate representative mitochondria. N: nucleus Scale bar = 10 µm (upper) or 500 nm (lower). (TIF) [file pone.0016054.s010.tif]

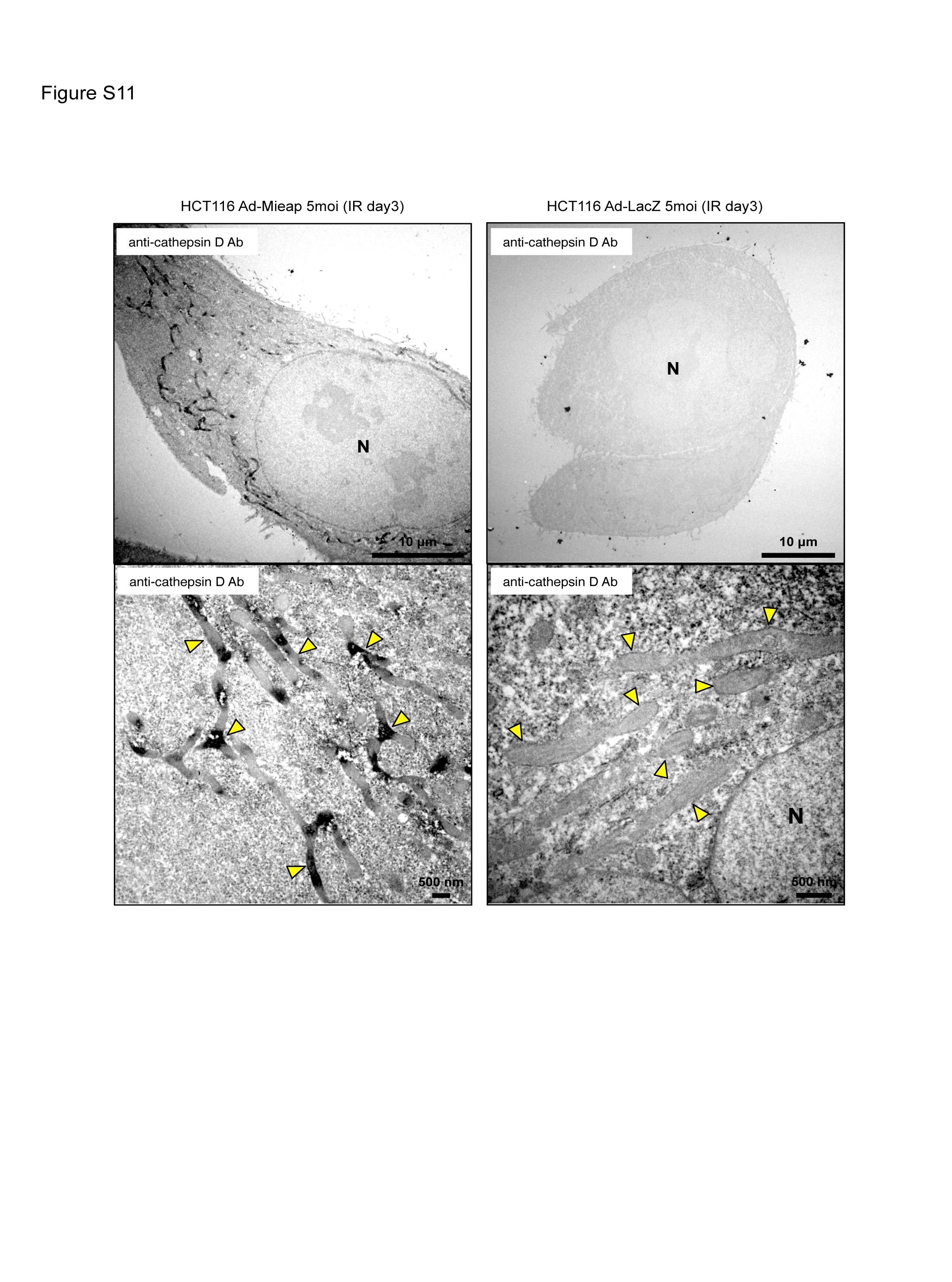

Supplement: Figure S11 — Pre-embedding immunoelectron microscopic analysis on cathepsin D. The Ad-LacZ and Ad-Mieap infected cells of HCT116 were subjected to pre-embedding immunoelectron microscopic analysis using DAB and anti-cathepsin D antibody on day 3 after IR. The representative images are shown. Arrowheads (yellow) indicate representative mitochondria. N: nucleus Scale bar = 10 µm (upper) or 500 nm (lower). (TIF) [file pone.0016054.s011.tif]

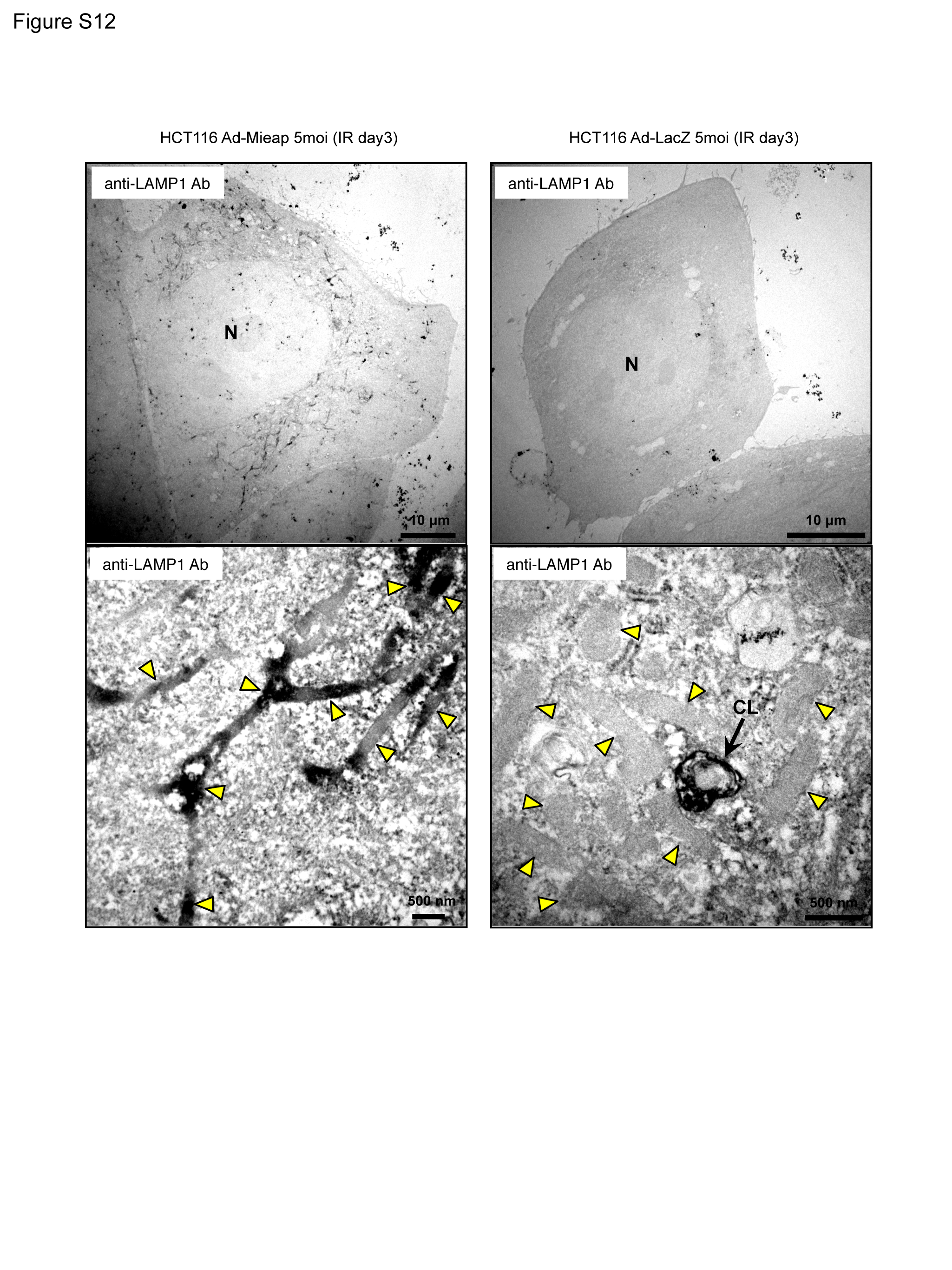

Supplement: Figure S12 — Pre-embedding immunoelectron microscopic analysis on LAMP1. The Ad-LacZ and Ad-Mieap infected cells of HCT116 were subjected to pre-embedding immunoelectron microscopic analysis using DAB and anti-LAMP1 antibody on day 3 after IR. The representative images are shown. Arrowheads (yellow) indicate representative mitochondria. N: nucleus CL: cytoplasmic lysosome Scale bar = 10 µm (upper) or 500 nm (lower). (TIF) [file pone.0016054.s012.tif]

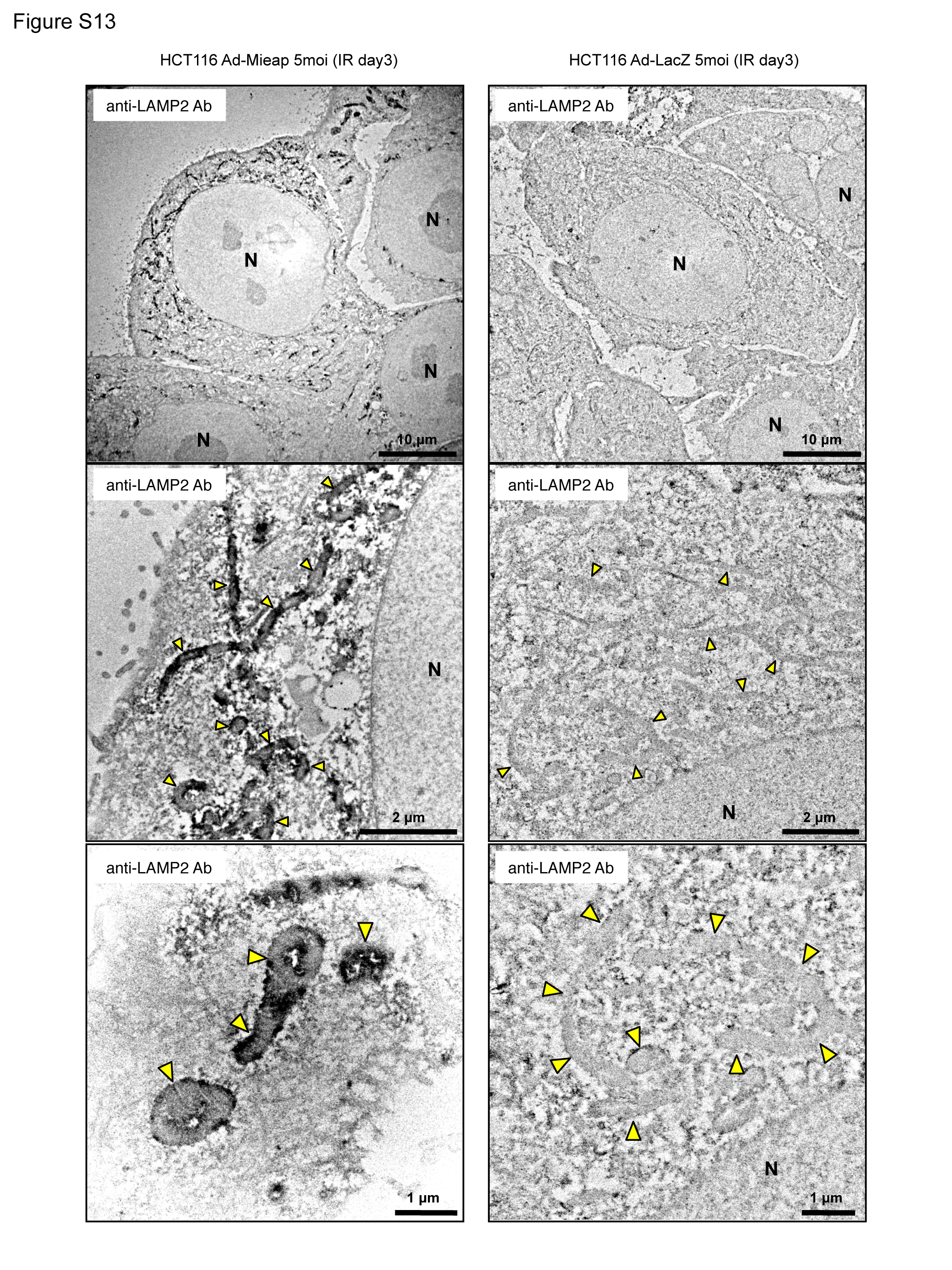

Supplement: Figure S13 — Pre-embedding immunoelectron microscopic analysis on LAMP2. The Ad-LacZ and Ad-Mieap infected cells of HCT116 were subjected to pre-embedding immunoelectron microscopic analysis using DAB and anti-LAMP2 antibody on day 3 after IR. The representative images are shown. Arrowheads (yellow) indicate representative mitochondria. N: nucleus Scale bar = 10 µm (upper), 2 µm (middle), or 1 µm (lower). (TIF) [file pone.0016054.s013.tif]

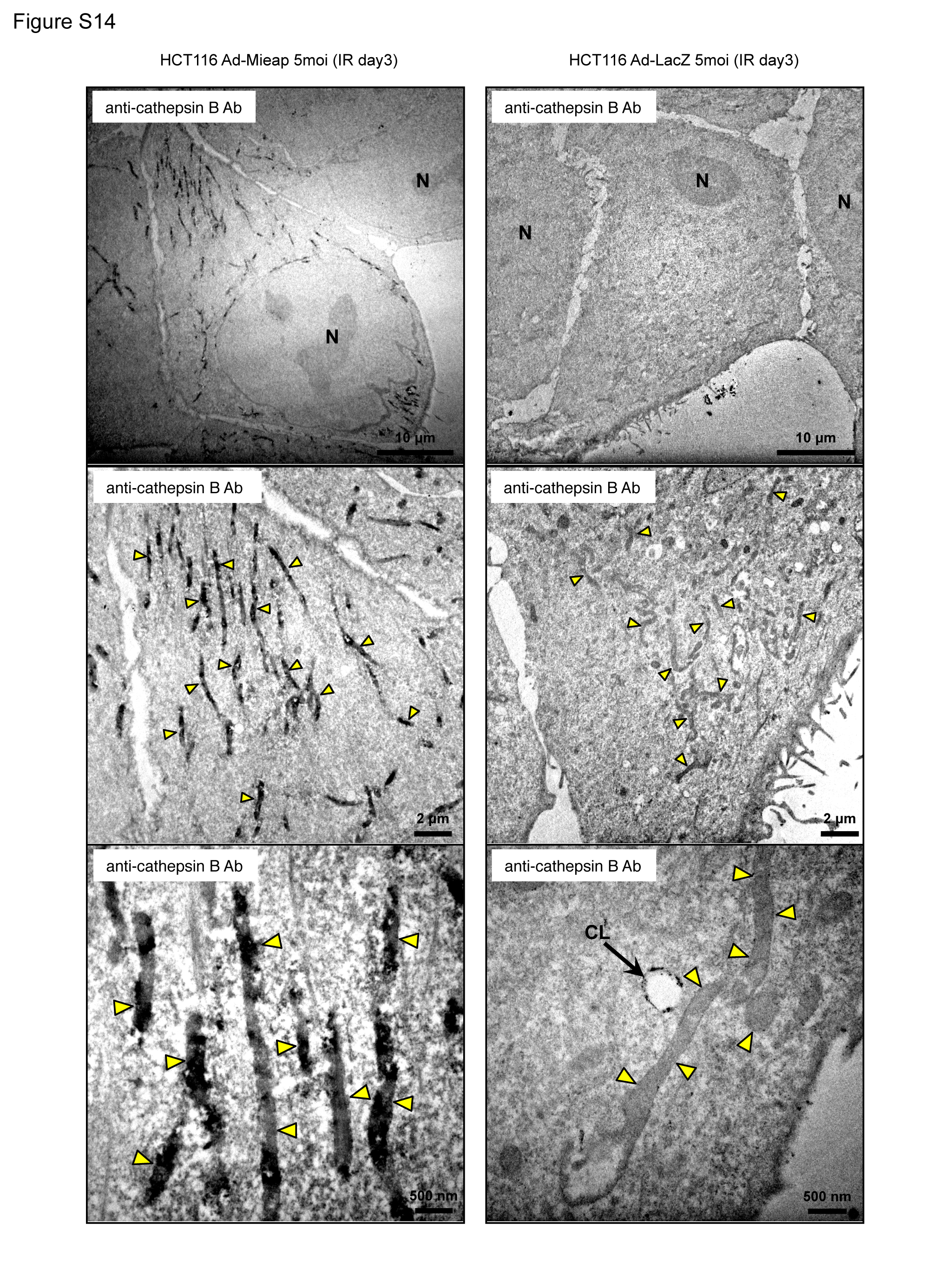

Supplement: Figure S14 — Pre-embedding immunoelectron microscopic analysis on cathepsin B. The Ad-LacZ and Ad-Mieap infected cells of HCT116 were subjected to pre-embedding immunoelectron microscopic analysis using DAB and anti-cathepsin B antibody on day 3 after IR. The representative images are shown. Arrowheads (yellow) indicate representative mitochondria. N: nucleus CL: cytoplasmic lysosome Scale bar = 10 µm (upper), 2 µm (middle), or 500 nm (lower). (TIF) [file pone.0016054.s014.tif]

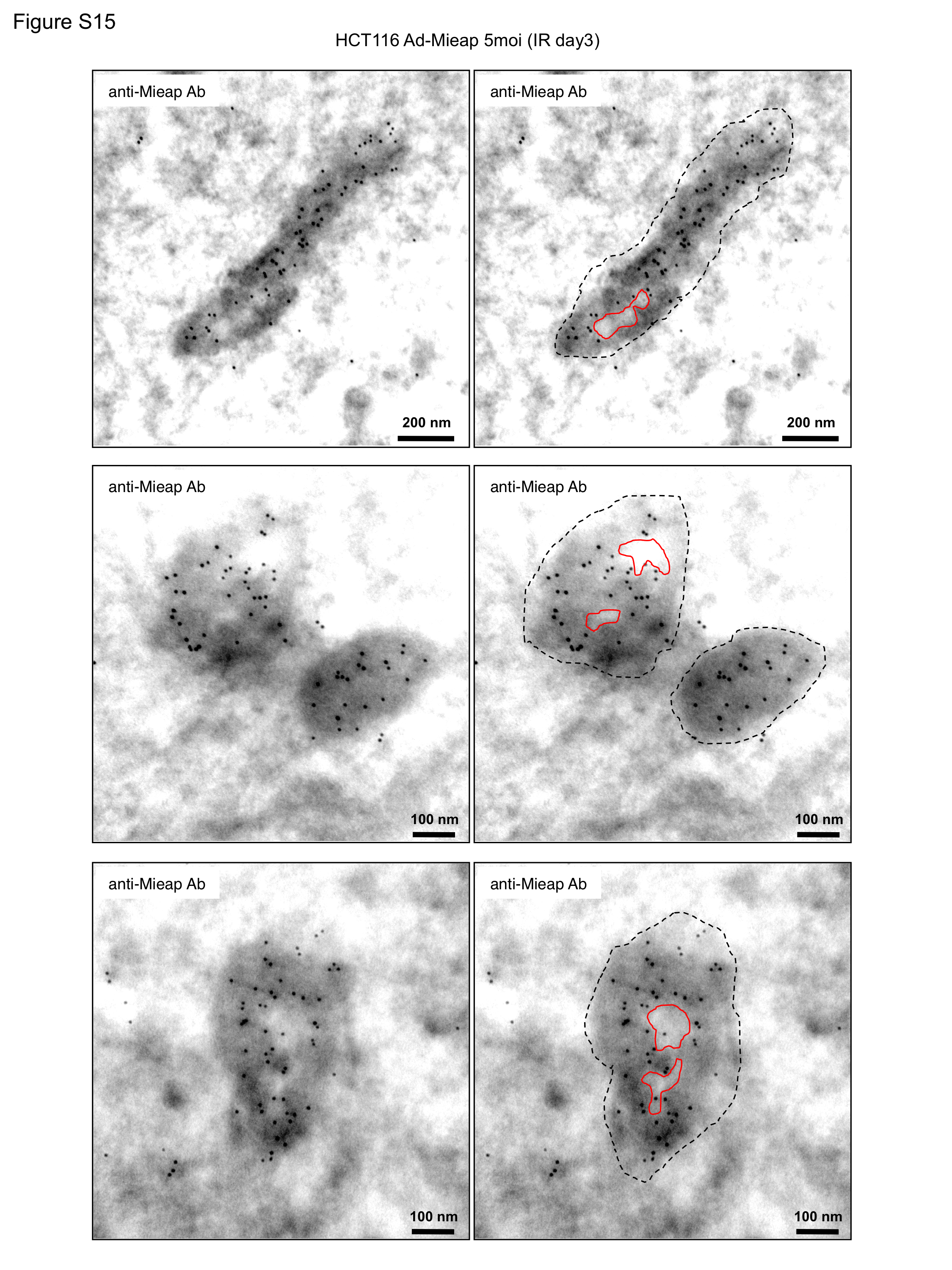

Supplement: Figure S15 — Post-embedding immunoelectron microscopic analysis on Mieap. The Ad-LacZ and Ad-Mieap infected cells of HCT116 were subjected to post-embedding immunoelectron microscopic analysis using gold particles and anti-Mieap antibody on day 3 after IR. The representative images are shown. The broken black line indicates the mitochondrial region. The red line indicates the mitochondrial cristae region. Scale bar = 100 nm or 200 nm. (TIF) [file pone.0016054.s015.tif]

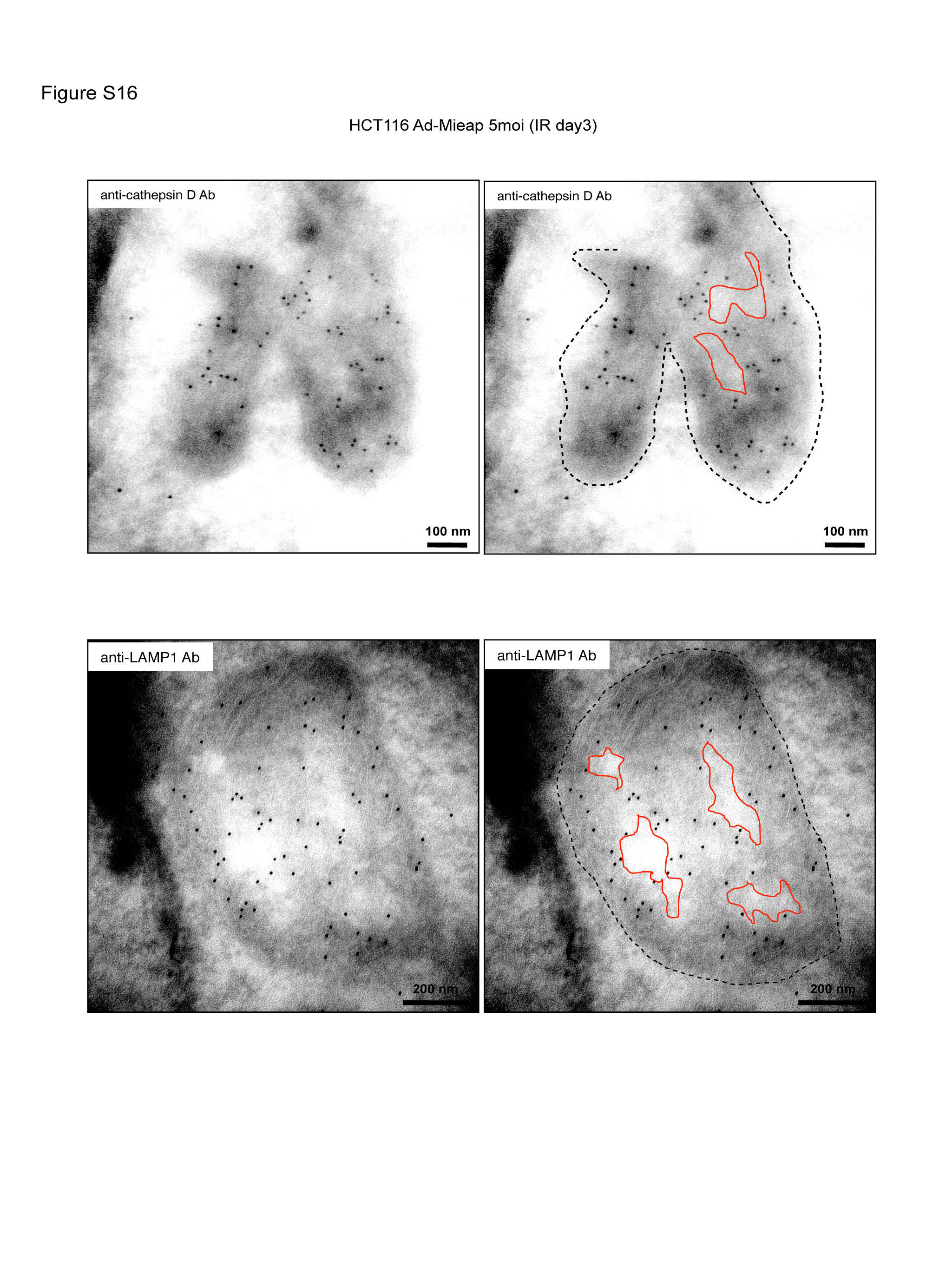

Supplement: Figure S16 — Post-embedding immunoelectron microscopic analysis on cathepsin D and LAMP1. The Ad-LacZ and Ad-Mieap infected cells of HCT116 were subjected to post-embedding immunoelectron microscopic analysis using gold particles, anti-cathepsin D antibody, and anti-LAMP1 antibody on day 3 after IR. The representative images are shown. The broken black line indicates the mitochondrial region. The red line indicates the mitochondrial cristae region. Scale bar = 100 nm or 200 nm. (TIF) [file pone.0016054.s016.tif]

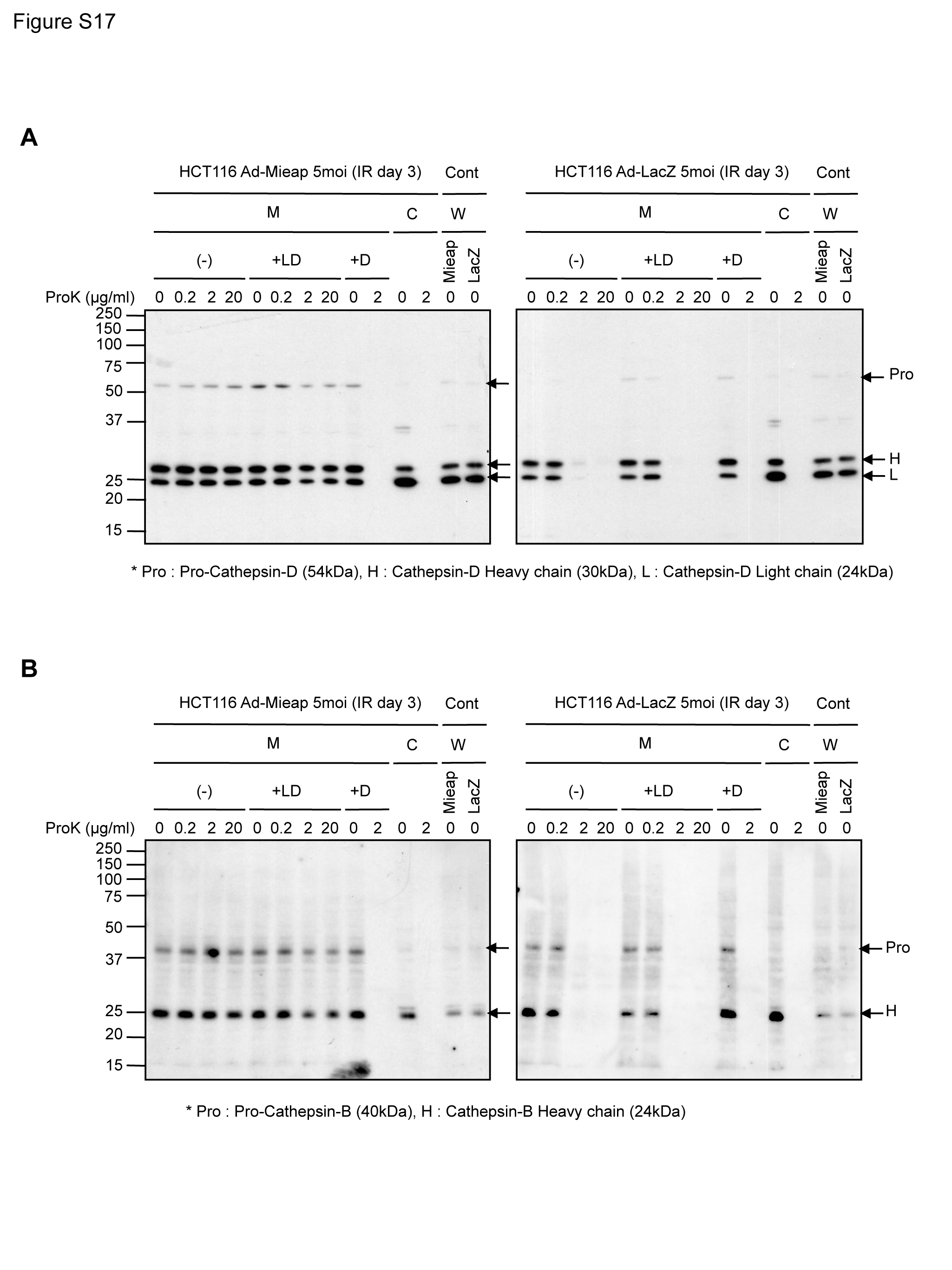

Supplement: Figure S17 — Whole image of western blot analysis on intramitochondrial and cytoplasmic proteins of cathepsin D and cathepsin B. The whole image of western blot analysis on intramitochondrial and cytoplasmic proteins of cathepsin D (A) and cathepsin B (B) in Figure 7 is shown. (TIF) [file pone.0016054.s017.tif]
